# Spreading of Alzheimer tau seeds is enhanced by aging and template matching with limited impact of amyloid-β

**DOI:** 10.1016/j.jbc.2021.101159

**Published:** 2021-09-02

**Authors:** Sarah Helena Nies, Hideyuki Takahashi, Charlotte S. Herber, Anita Huttner, Alison Chase, Stephen M. Strittmatter

**Affiliations:** 1 Cellular Neuroscience, Neurodegeneration and Repair Program, Departments of Neurology and Neuroscience, Yale School of Medicine, New Haven, Connecticut, USA; 2 Graduate School of Cellular and Molecular Neuroscience, University of Tübingen, Tübingen, Germany; 3 Department of Pathology, Yale School of Medicine, New Haven, Connecticut, USA

**Keywords:** Alzheimer's disease, tau protein, amyloid-beta, transgenic mice, stereotactic injection, Aβ, amyloid-β, Aβo, Aβ oligomer, AD, Alzheimer's disease, APP, amyloid precursor protein, AZD, AZD0530, DAB, 3,3'-diaminobenzidine, ddH_2_O, double-distilled water, DIV, days *in vitro*, DKI, double knock-in mice, EC, entorhinal cortex, GFAP, glial fibrillary acidic protein, hTau, humanized tau, IHC, immunohistochemistry, KI, knock-in, MAPT, microtubule-associated protein tau, NFT, neurofibrillary tangle, NP, neuritic plaque, NT, neuropil thread, PGRN, progranulin, ROI, region of interest, RSA, retrosplenial area, RT, room temperature, TBST, Tris-buffered saline + 0.1% Tween-20 detergent, ThioS, thioflavin S, WB, Western blotting

## Abstract

In Alzheimer's disease (AD), deposition of pathological tau and amyloid-β (Aβ) drive synaptic loss and cognitive decline. The injection of misfolded tau aggregates extracted from human AD brains drives templated spreading of tau pathology within WT mouse brain. Here, we assessed the impact of Aβ copathology, of deleting loci known to modify AD risk (*Ptk2b*, *Grn*, and *Tmem106b*) and of pharmacological intervention with an Fyn kinase inhibitor on tau spreading after injection of AD tau extracts. The density and spreading of tau inclusions triggered by human tau seed were unaltered in the hippocampus and cortex of *APPswe/PSEN1ΔE9* transgenic and *App*^*NL-F/NL-F*^ knock-in mice. In mice with human tau sequence replacing mouse tau, template matching enhanced neuritic tau burden. Human AD brain tau-enriched preparations contained aggregated Aβ, and the Aβ coinjection caused a redistribution of Aβ aggregates in mutant AD model mice. The injection-induced Aβ phenotype was spatially distinct from tau accumulation and could be ameliorated by depleting Aβ from tau extracts. These data suggest that Aβ and tau pathologies propagate by largely independent mechanisms after their initial formation. Altering the activity of the Fyn and Pyk2 (*Ptk2b*) kinases involved in Aβ-oligomer–induced signaling, or deleting expression of the progranulin and TMEM106B lysosomal proteins, did not alter the somatic tau inclusion burden or spreading. However, mouse aging had a prominent effect to increase the accumulation of neuritic tau after injection of human AD tau seeds into WT mice. These studies refine our knowledge of factors capable of modulating tau spreading.

Advancing our understanding of the molecular causes underlying Alzheimer's disease (AD), especially amyloid-β (Aβ) and tau pathologies, becomes more important as aging populations cause a continuous rise in AD cases globally (1, 2).

In the human brain, the microtubule-associated protein tau (*MAPT*) gene encodes six isoforms of tau that differ in the number of amino-terminal inserts (0N, 1N, and 2N) and carboxy-terminal repeat sequences (3R or 4R) (3, 4). In disease, tau becomes hyperphosphorylated and is deposited intracellularly as neurofibrillary tangles (NFTs), neuritic plaques (NPs), and neuropil threads (NTs) (4, 5, 6). NFT deposition correlates better than Aβ plaque load with progression of neuronal loss and memory deficits in patients with AD (7, 8). Different tauopathies are characterized by the phosphorylation of distinct tau residues, the type of inclusions, and the cell type in which they occur (neurons, astrocytes, or oligodendrocytes) (9). This has led to the theory of prion-like tau transmission where different tau seeds serve as templates to corrupt endogenous tau into misfolding, resulting in prion-like tau strains with distinct spreading patterns (10, 11). In AD, tau deposits are restricted to neurons, and both 3R and 4R tau are recruited to NFTs (4, 9). The spreading of NFTs can occur along neuronal connections and is thought to be mediated through *trans*-synaptic release of tau (12, 13, 14, 15, 16, 17, 18). Cortical inclusions first appear in the *trans*-entorhinal cortex (EC) and then spread *via* the EC to the hippocampus and other brain regions (19, 20, 21, 22, 23). The extent to which cell-autonomous intracellular tau aggregation and transneuronal spreading are dependent on one another remains unclear. The balance of *de novo versus* templated misfolding of tau in AD brain is also ill defined. In this context, there is ongoing debate regarding which aspects of cell biology in AD might alter the pattern and magnitude of tau spreading.

One mechanism proposed to modify tau aggregation and spreading is autophagy, which is dysregulated in several neurodegenerative diseases (24, 25). In AD, neurons likely remove some deposits of hyperphosphorylated tau and aggregated Aβ through autophagy/lysosomal degradation, and inhibition of lysosomal function increases intracellular tau and Aβ accumulation (25, 26). Progranulin (PGRN) and TMEM106B are lysosomal proteins implicated in many neurodegenerative disorders including frontotemporal lobar degeneration and AD (27, 28, 29, 30). Importantly, complete loss of PGRN causes neuronal ceroid lipofusinosis, a lysosomal storage disorder (31). Lysosome enzyme dysregulation and accumulation of lipofuscin were also found in PGRN-deficient mice (32, 33, 34). Interestingly, reduction of PGRN has been recently associated with increased cerebrospinal fluid total tau in humans and tau phosphorylation in mice (35, 36, 37). TMEM106B reduction or loss has been reported to cause multiple lysosomal abnormalities, including impaired acidification and dysregulated trafficking of lysosomes in mouse neurons (32, 38, 39, 40).

There is conflicting evidence as to what role Aβ aggregation plays in enhancing tau pathology at various stages of AD (41, 42, 43, 44, 45, 46, 47, 48). Amyloid precursor protein (APP) is processed by β- and γ-secretases to generate Aβ monomers, which form fibrillar Aβ oligomers (Aβos) that can aggregate further and be deposited as extracellular Aβ plaques (49, 50, 51, 52). Aβos are thought to be the most toxic species since they can induce signaling cascades leading to synaptic dysfunction, neuronal loss, and cognitive decline (53, 54, 55). In contrast, Aβ plaques induce neuroinflammation (56) but might also have a neuroprotective function by sequestering Aβo (50, 57). Aβ accumulation is likely causative for early onset familial AD and also thought to play a pivotal role in sporadic late-onset AD, since Aβ plaque deposition generally precedes the emergence of pathological tau accumulation, such that Aβ may initiate tau pathology (58, 59). One possible connection between Aβo and tau is a signaling cascade wherein Aβo bind to the prion protein receptor (PrP^C^), which then interacts with the metabotropic glutamate receptor 5, leading to activation of Fyn and Pyk2 (*Ptk2b*) kinases (60, 61, 62, 63, 64). Both kinases have been reported to interact with and phosphorylate tau directly (65, 66, 67, 68) and to activate glycogen synthase kinase 3β, one of the most well-characterized tau kinases in AD (69, 70, 71).

Here, we explored the impact that mechanisms altering intracellular tau hyperphosphorylation and aggregation have on tau spreading. Therefore, we injected tau seeds extracted from human AD brain into mice. We investigated the impact of Aβ pathology on tau spreading by injecting the *APPswe/PSEN1ΔE9* and *App*^*NL-F/NL-F*^ mouse models. Furthermore, we explored how reducing the activation of potential tau kinases impacted tau spreading by injecting *Ptk2b*^*−/−*^ mice as well as pharmacologically inhibiting Fyn kinase. Finally, we investigated the effects of perturbing lysosomal regulation on tau spreading by injecting *Grn*^*−/−*^ and *Tmem106b*^*−/−*^ mice. We find that the primary factors titrating tau spread are template matching and mouse aging.

## Results

### Generating tau extracts from neurologically intact and AD subject brains

**Figure 1:**
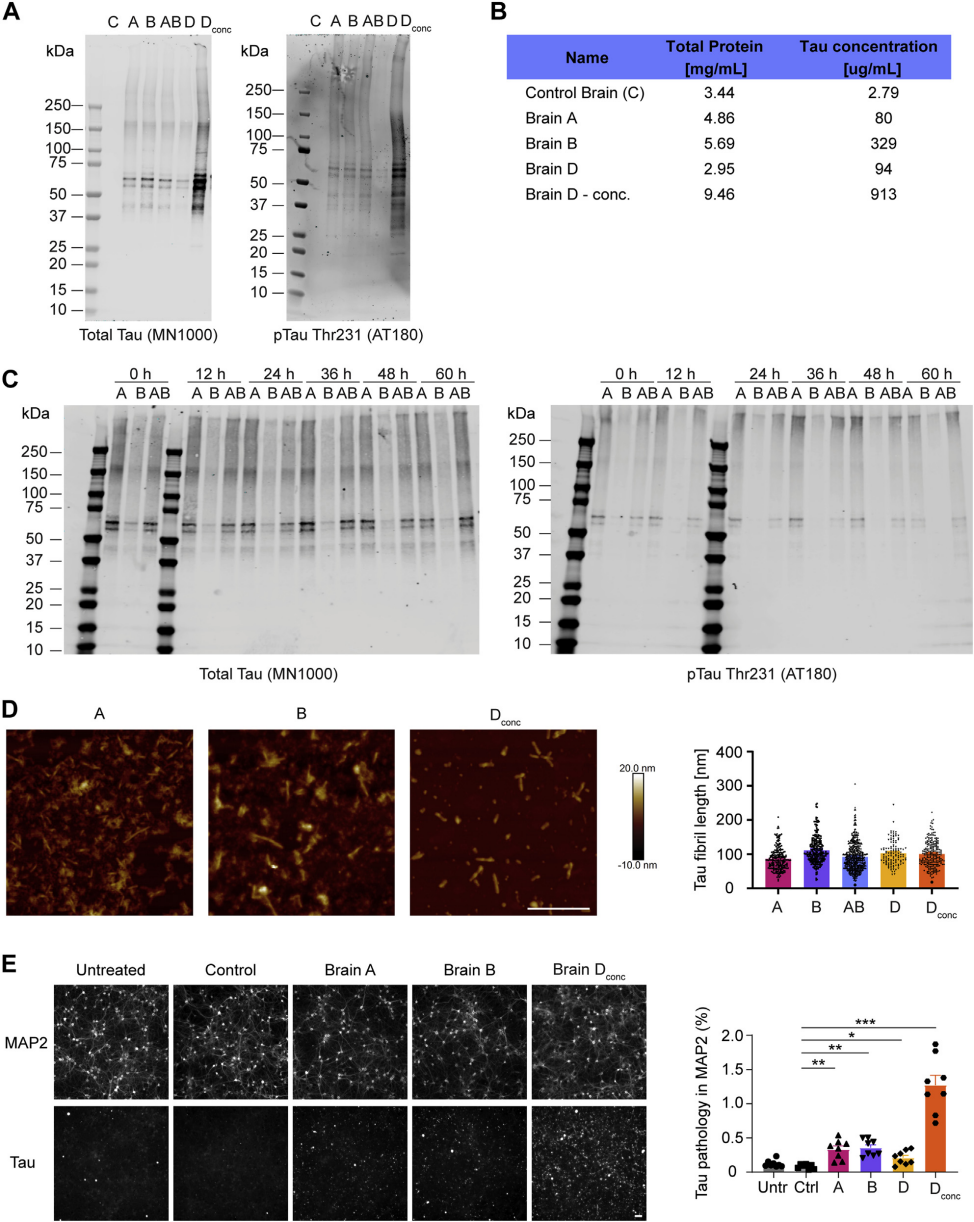
**Characterization of tau fibrils extracted from human AD subjects.***A*, immunoblots for total tau (HT7) and pTau-Thr231 (AT180) of the tau extracts used in this study (samples were as follows: C: control, A: brain A, B: brain B, AB: 1:1 mixture of brain A and B extracts, D: brain D, and D_conc_: 10× concentrated brain D). *B*, overview of total protein and total tau concentration in each extract. Total protein concentration was evaluated by measuring absorption at 280 nm on a spectrophotometer. Total tau concentration was calculated by comparing densitometric quantifications of total tau (HT7) immunoblots to a dilution curve of recombinant tau (2N4R isoform). *C*, immunoblots for total tau (HT7) and pTau-Thr231 (AT180) for samples from brain A, B, and a 1:1 mixture of brain A and B (AB) to assess the tau isoform distribution and phosphorylation over a 60-h time course with measurements taken every 12 h. *D*, tau extracts were diluted in PBS to a concentration of 5 μg/ml, and 5 × 5 μm images were taken by atomic force microscopy (the scale bar represents 0.5 μm). Fibril length was quantified using Gwyddion. N(A) = 219 fibrils from two images, N(B) = 260 fibrils from three images, N(AB) = 494 fibrils from three images, N(D) = 116 fibrils from two images, and N(D_conc_) = 225 fibrils from two images. *E*, primary mouse neurons were seeded at 50,000 to 75,000 density, treated with 0.25% (v/v) human tau extracts at DIV 7 and incubated until DIV 21 (the scale bar represents 50 μm). Cells were fixed in ice-cold methanol and stained for MAP2 and mouse tau (T49). Tau seeding was measured by quantifying the percent area occupied by aggregated mouse tau within MAP2-positive area using ImageJ. Statistics: Brown–Forsythe ANOVA test (*F* = 43.85; *p* < 0.0001) with Dunnett's multiple comparisons test to compare all groups to control-treated cells. N = 8 images per condition. ∗*p* < 0.05, ∗∗*p* < 0.01, ∗∗∗*p* < 0.005, and ∗∗∗∗*p* < 0.0001. AD, Alzheimer's disease; DIV, days *in vitro*.

To obtain tau extracts able to seed tau aggregation in WT mice, we followed a published tau purification protocol (18) with autopsy brain tissue from one neurologically intact (from now on called control) subject and three different AD subjects (see Fig. S1*A* for post-mortem information). To obtain concentrated brain D tau extracts, the final resuspension volume during the purification protocol was reduced to one quarter of the original amount. To assess the total tau and phospho-tau concentrations in extracts, we performed immunoblots with anti-total tau (HT7) and pTau (AT180) antibodies (Fig. 1*A*) and measured tau concentration by comparing tau extracts to a standard curve of recombinant 2N4R tau (Fig. 1*B*). Control subject–derived brain extract contained very low amounts of human tau while showing total protein concentration comparable to the extracts derived from AD subjects. The nonconcentrated AD subject-derived extracts showed minor variation in their tau concentrations. To provide a larger stock for mouse injections with the same tau preparation, tau extracted from brain A and B was combined in a 1:1 ratio for most mouse injections (from here on termed AB extract). Next, we sought to test if the phosphorylation and aggregation of this tau material would remain stable over a 60-h period when stored on ice. This was important to ensure that a cohort of mice injected over the course of up to 3 days would receive the same material. There were no significant changes in tau high–molecular-weight aggregates or phosphorylation when comparing samples from extracts stored at 4 °C every 12 h (Fig. 1*C*). We also characterized the length of our extracted tau fibrils using an atomic force microscope (Fig. 1*D*). We observed similar average fibril lengths in all tau extracts extracted from AD brains of 90 to 100 nm. To test the potency of our tau extracts *in vitro*, we treated days *in vitro* (DIV) 7 primary WT mouse neurons with 0.25% (v/v) tau extracts for 14 days and measured tau pathology within the area occupied by neurons (Fig. 1*E*). Treatment with control extract did not induce tau pathology, whereas all AD tau extracts caused significant increases in tau pathology. Of note, treating with about 10-fold higher concentration of tau (brain D_conc_) resulted in a nonlinear increase in tau pathology.

### Tau from different AD subjects generated somatic and neuritic inclusions in WT mice

**Figure 2:**
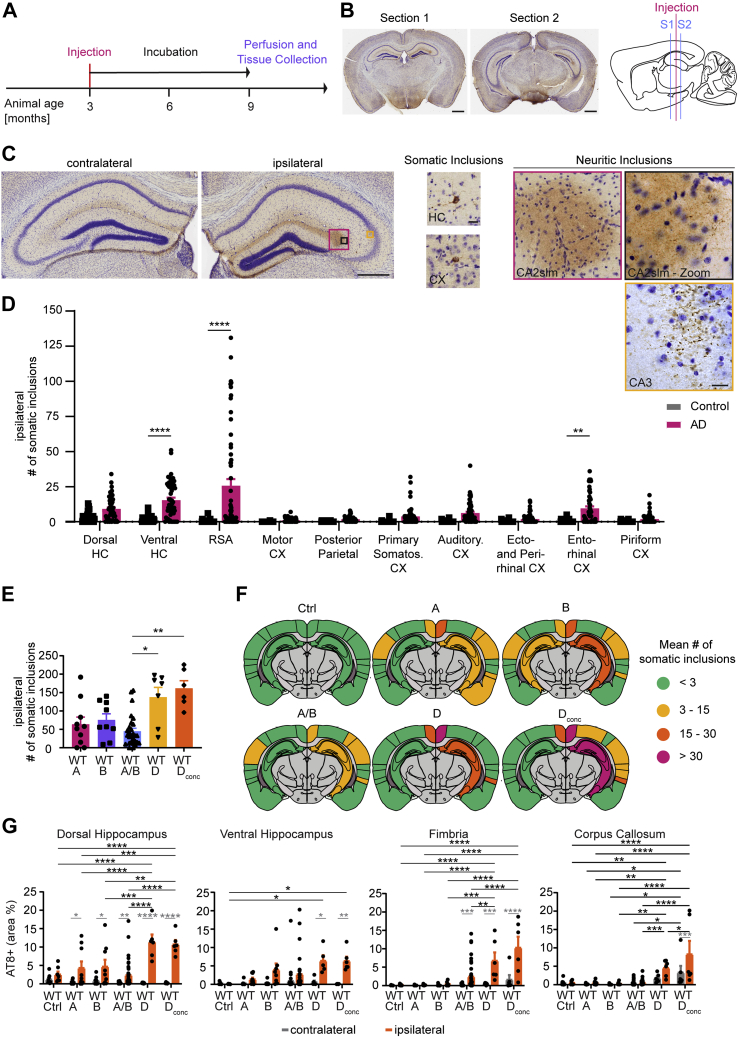
**Injecting tau extracts into WT mice results in tau deposition and spreading in hippocampus and cortex.***A*, experimental time line of mouse injections. Mice were injected unilaterally (*right hemisphere*) at 3 months of age with either control or AD brain tau extract. Per animal, 5 μl of tau extract were injected distributed over two injection sites (2.5 μl/site). After injection, mice were housed under regular conditions for 6 months and then killed by perfusion. *B*, example pictures of AT8b-DAB + Nissl stain sections that were analyzed and sagittal schematic indicating injection site in *pink* and analyzed section location in *blue*. Section 1 is located anterior (from Bregma: ML +2.0 mm, AP −2.0) to the injection sites (from Bregma: anterior–posterior −2.5 mm; medial–lateral 2 mm; dorsoventral −2.4 mm [for hippocampus] and dorsoventral −1.4 mm [for cortex]), section 2 is located posterior (ML +2.0 mm, AP −2.9) to the injection sites (the scale bar represents 1 mm). *C*, from *left* to *right*: magnified images of section 1 contralateral and ipsilateral hippocampus (the scale bar represents 0.5 mm). Example images of somatic inclusions quantified in this study (the scale bar represents 25 μm). Example images of neuritic inclusions quantified in this study (the scale bar represents 25 μm). *D*, mean number of somatic inclusions per brain region on the ipsilateral hemisphere of WT animals injected with control or AD tau extracts. Inclusions were counted manually in ImageJ with the Cell Counter Tool. Statistics: Ordinary two-way ANOVA test (interaction: *F*(9, 841) = 8.431, *p* < 0.0001; row factor: *F*(9, 841) = 9.852, *p* < 0.0001; column factor: *F*(1, 841) = 72.28, *p* < 0.0001) with Sidak's multiple comparisons test. N represents individual animals. N(control) = 27 and 28, N(AD) = 60. ∗∗*p* < 0.01, ∗∗∗∗*p* < 0.0001. *E*, mean number of somatic inclusions on the ipsilateral hemisphere in animals injected with tau extracts extracted from different AD brains. Statistics: Kruskal–Wallis test (approximate *p*: 0.0008, Kruskal–Wallis statistic: 18.91) with Dunn's multiple comparisons. N represents individual animals. N(A) = 10, N(B) = 9, N(AB) = 28, N(D) = 7, and N(D_conc_) = 6. ∗*p* < 0.05, ∗∗*p* < 0.01. *F*, coronal section schematics of mean somatic inclusion burden in ipsilateral and contralateral brain regions dependent on injected tau extract. Brain regions not analyzed are depicted in *gray*. Ipsilateral hemisphere is on the *right*. *G*, area occupied by neuritic inclusions in four regions of animals injected with tau extracts from different AD brains. Statistics: Ordinary two-way ANOVA (for dorsal hippocampus—interaction: *F*(5, 144) = 9.266, *p* < 0.0001; row factor: *F*(5, 144) = 9.041, *p* = 0.0001; column factor: *F*(1, 144) = 95.96, *p* < 0.0001; for ventral hippocampus—interaction: *F*(5, 121) = 2.191, *p* = 0.0596; row factor: *F*(5, 121) = 2.120, *p* = 0.0675; column factor: *F*(1, 121) = 21.06, *p* < 0.0001; for fimbria—interaction: *F*(5, 144) = 6.743, *p* < 0.0001; row factor: *F*(5, 144) = 11.41, *p* = 0.0001; column factor: *F*(1, 144) = 33.45, *p* < 0.0001; for corpus callosum—interaction: *F*(5, 137) = 3.679, *p* = 0.0037; row factor: *F*(5, 137) = 18.48, *p* < 0.0001; column factor: *F*(1, 137) = 12.78, *p* = 0.0005) with Sidak's multiple comparisons test. In *gray*: comparing ipsilateral and contralateral hemisphere for the same injected extract. In *black*: comparing ipsilateral values of different tau extract–injected groups. N represents individual animals. N(control) = 8 and 9, N(A) = 9 and 10, N(B) = 8 and 9, N(AB) = 30 to 39, N(D) = 6 and 7, and N(D_conc_) = 6. ∗*p* < 0.05, ∗∗*p* < 0.01, ∗∗∗*p* < 0.005, and ∗∗∗∗*p* < 0.0001. AD, Alzheimer's disease.

To ascertain if our tau extracts showed seeding activity *in vivo*, we injected WT animals as described previously (18). Tau extracts were injected into the hippocampus and overlying cortex of one hemisphere at 3 months of age (Fig. 2, *A* and *B*). After a 6-month waiting period, we analyzed the number of somatic inclusions in the hippocampus and cortex in two standardized sections, as well as the percent area occupied by neuritic inclusions in hippocampus, fimbria, and corpus callosum (Fig. 2, *B* and *C*). To be counted as a somatic inclusion, AT8-positive tau deposition was required to be present in a neuronal cell soma and outline the cell body. The somatic inclusions are likely to include deposition in pre-NFT and NFT stages. We did not assess mature NFTs separately by silver stain or electron microscopy. Neuritic tau inclusions counted by our thresholding include both very fine NTs within the hippocampus and slightly larger threads with the occasional round inclusion in white matter tracts (Fig. 2*C*). Since we injected tau from patients with AD, it is likely that NTs detected in white matter tracts are within neurites, not oligodendrocytes. In the dorsal and ventral hippocampus, the threshold to identify the percent area occupied by neuritic inclusions also recognizes and includes somatic inclusions, but the somatic fraction constitutes less than 5% of the total neuritic inclusion area measured.

We monitored mouse health by monthly weighing (Fig. S1*B*) and detected no adverse effects of AD extract injection on mouse body mass. When comparing the number of somatic inclusions on the ipsilateral hemisphere of control or AD extract–injected animals in each brain region (Fig. 2*D*), tau inclusions were only found to be seeded at significant levels by AD extracts in select brain regions (ventral hippocampus, retrosplenial area [RSA], and EC). A similar pattern was observed in the contralateral hemisphere (Fig. S2*A*), where only RSA and auditory cortex showed significantly higher levels of somatic tau inclusions. For AD-injected animals, the number of somatic inclusions in each contralateral region was lower than the one in their ipsilateral counterpart. Since tau fibrils were extracted from several different AD brains, we sought to evaluate whether injecting material from different subjects would alter the number of somatic inclusions per animal. Extracts A (80 μg tau protein/ml), B (329 μg tau protein/ml), AB (204 μg tau protein/ml), and D (94 μg tau protein/ml) were injected into mice directly, whereas the concentrated brain D extract was diluted in sterile PBS to a concentration of 500 μg/ml before injection. Increasing (brain D concentrated) or decreasing (brain A and D) the amount of injected Tau did not translate into a proportional change in tau seeding in WT mice. Instead, the main difference in seeding was observed between tau extracts derived from different brains, with brain D resulting in more numerous tau inclusions (Fig. 2*E* and Fig. S2*B*). The regional distribution of tau inclusions between the different tau extracts though remained largely unchanged (Fig. 2*F*), with ventral hippocampus, RSA, and EC on the ipsilateral hemisphere showing the most inclusions. The extract used to inject most cohorts was AB, and the tau spreading pattern for this extract remained the same throughout the WT animals of different cohorts (Fig. S1, *C* and *D*). Furthermore, the neuritic tau burden in WT animals was elevated in all measured regions for brain D or D_conc_ injected animals compared with brain A, B, or AB injected ones (Fig. 2*G*). Altogether, these data are consistent with previous observations (18).

### Aβ accumulation did not alter tau inclusion burden, but human tau template increased neuritic inclusions

Having established that AD tau injection leads to tau accumulation and spreading in WT animals, we investigated whether the presence of Aβ deposition would impact tau spreading using the *APPswe/PSEN1ΔE9* transgenic (APP mice) and *App*^*NL-F/NL-F*^ knock-in (KI) mouse models. APP mice develop Aβ plaques between 4 and 6 months of age. This mouse model is well established but has the caveat of Aβ overexpression *via* the transgene array. *App*^*NL-F/NL-F*^ KI mice develop the plaques around 6 months of age. We intercrossed the KI mice with a mouse containing humanized tau (hTau) to generate double homozygous hTau-*App*^*NL-F/NL-F*^ mice (double KI [DKI]) (72). To discern if changes in tau spreading were caused by humanized Aβ or hTau, we also injected hTau animals not carrying the *App*^*NL-F/NL-F*^ gene with tau extracts.

**Figure 3:**
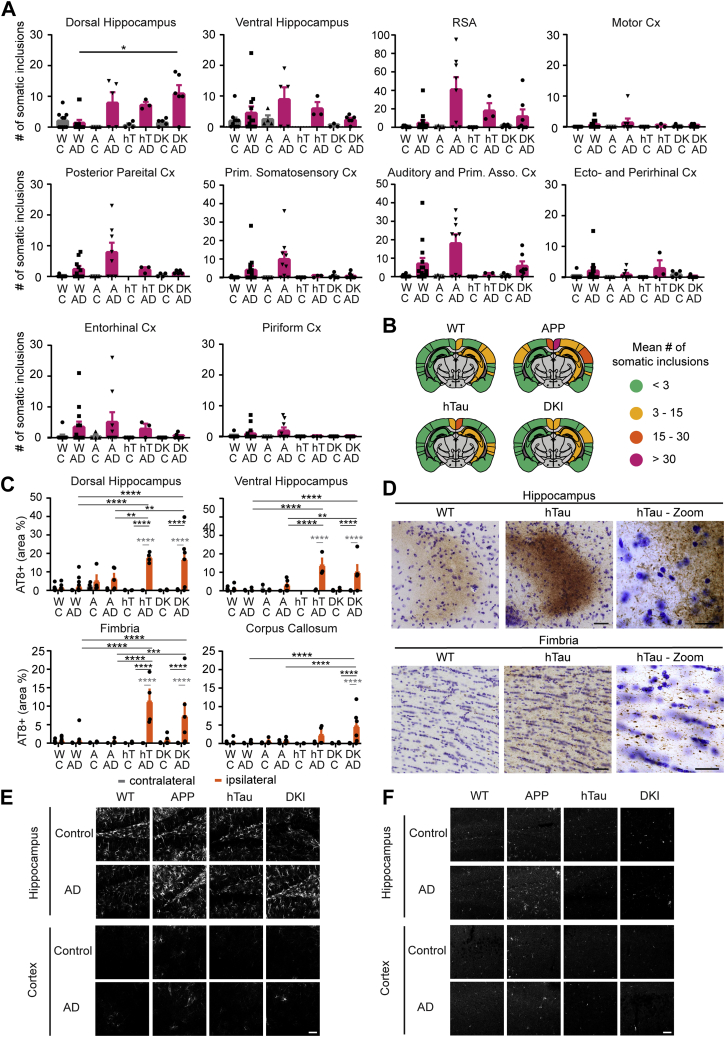
**Aβ copathology has no impact on tau inclusion burden or spreading, but the presence of humanized tau (hTau) enhances tau deposition.***A*, mean number of somatic inclusions in different ipsilateral brain regions of animals injected with tau extracts extracted from control or AD brains. C = control, W = WT, A = APP, hT = hTau, and DK = DKI. Statistics: Kruskal–Wallis test (for dorsal hippocampus (HC)—approximate *p* value = 0.0155, Kruskal–Wallis statistic = 10.39; for ventral HC—approximate *p* value = 0.4352, Kruskal–Wallis statistic = 2.730; for RSA—approximate *p* value = 0.0155, Kruskal–Wallis statistic = 10.39; for motor cortex (Cx)—approximate *p* value = 0.8045, Kruskal–Wallis statistic = 0.9867; for posterior parietal—approximate *p* value = 0.6084, Kruskal–Wallis statistic = 1.83; for primary somatosensory Cx—approximate *p* value = 0.1169, Kruskal–Wallis statistic = 5.894; for auditory Cx—approximate *p* value = 0.2602, Kruskal–Wallis statistic = 4.012; for ectorhinal and perirhinal Cx—approximate *p* value = 0.1885, Kruskal–Wallis statistic = 4.781; for entorhinal Cx—approximate *p* value = 0.4783, Kruskal–Wallis statistic = 2.484; for piriform Cx—approximate *p* value = 0.1606, Kruskal–Wallis statistic = 5.158) with Dunn's multiple comparisons test. N represents individual animals. N(WT-C) = 9 and 10, N(WT-AD) = 14 and 15, N(APP-C) = 4, N(APP-AD) = 8 and 9, N(hT-C) = 4, N(hT-AD) = 3, N(DK-C) = 5, and N(DK-AD) = 7. ∗*p* < 0.05. *B*, schematics of mean somatic inclusion burden of AD tau extract–injected animals dependent on mouse genotype. Brain regions not analyzed are depicted in *gray*. Ipsilateral hemisphere is on the *right*. *C*, area occupied by neuritic inclusions in four brain regions of animals injected with control or AD tau extracts. Statistics: Ordinary two-way ANOVA (for dorsal hippocampus—interaction: *F*(7, 88) = 8.073, *p* < 0.0001; row factor: *F*(7, 88) = 7.714, *p* < 0.0001; column factor: *F*(1, 88) = 38.19, *p* < 0.0001; for ventral hippocampus—interaction: *F*(6, 62) = 8.017, *p* < 0.0001; row factor: *F*(6, 62) = 7.733, *p* < 0.0001; column factor: *F*(1, 62) = 30.04, *p* < 0.0001; for fimbria—interaction: *F*(7, 90) = 7.293, *p* < 0.0001; row factor: *F*(7, 90) = 7.084, *p* < 0.0001; column factor: *F*(1, 90) = 24.14, *p* < 0.0001; for corpus callosum—interaction: *F*(7, 83) = 4.807, *p* = 0.0001; row factor: *F*(7, 83) = 4.837, *p* = 0.0001; column factor: *F*(1, 83) = 13.40, *p* = 0.0004) with Sidak's multiple comparisons test. In *gray*: comparing ipsilateral and contralateral hemisphere for the same injected extract. In *black*: comparing ipsilateral values of different tau extract–injected groups. N represents individual animals. N(WT-C) = 9 and 10, N(WT-AD) = 14 and 15, N(APP-C) = 4, and N(APP-AD) = 5 to 9, N(hT-C) = 0 to 4, N(hT-AD) = 3 and 4, N(DK-C) = 5, and N(DK-AD) = 5 and 6. ∗*p* < 0.05, ∗∗*p* < 0.01, ∗∗∗*p* < 0.005, and ∗∗∗∗*p* < 0.0001. *D*, representative images of neuritic inclusions in fimbria and hippocampus (the scale bars represent 50 μm [hTau] and 25 μm [hTau-Zoom]). *E*, representative images of GFAP staining in the ipsilateral hemisphere hippocampus (DG) and cortex (layers I–III). Images were taken with a 20× objective (the scale bar represents 50 μm), for quantification, see Fig. S2*D*. *F*, representative images of CD68 staining in the ipsilateral hemisphere hippocampus (DG) and cortex (layers I–III). Images were taken with a 20× objective (the scale bar represents 50 μm), for quantification, see Fig. S2*E*. AD, Alzheimer's disease; APP, amyloid precursor protein; DKI, double KI; RSA, retrosplenial area.

After AD tau extract injection into these different Aβ plaque containing mice, we observed only a limited and nonsignificant trend to increase the number of somatic tau inclusions in the cortex of AD tau-injected mice compared with WT mice. In the hippocampus, only DKI animals injected with AD tau extract showed a significant difference in the dorsal hippocampus (Fig. 3, *A* and *B*). We observed no change in tau-positive NPs near amyloid plaques for either the APP or the DKI model (Fig. S3*D*). In contrast, the amount of neuritic inclusions was significantly increased in mice carrying hTau for all measured regions (Fig. 3, *C* and *D*). Adding *App*^*NL-F/NL-F*^ to the hTau genotype (DKI mice) did not cause further increase in the neuritic tau inclusion burden. Under these conditions, the presence of Aβ accumulation does not alter tau spreading. The findings support the hypothesis that misfolded human tau seeds can template hTau more efficiently than murine tau. We next sought to explore if there might be altered glial reaction, which in turn might alter tau aggregation in neurons. We stained brain sections for glial fibrillary acidic protein (GFAP) (Fig. 3*E* and Fig. S2*D*) and CD68 (Fig. 3*F* and Fig. S2*E*), which are astrocytic and microglial markers, respectively, and analyzed the percent area occupied by each staining in the hippocampus and cortex. The only injection-dependent difference detected was for CD68 signal in the hippocampus of AD extract–injected animals. In all other groups, the AD brain injection did not alter immunoreactivity of these glial markers. Thus, AD tau aggregate induction of spreading tau inclusions in these mice is enhanced by matching the template but is largely independent on Aβ pathology or gliosis.

### Aβ present in AD tau extracts induces Aβ redistribution in AD model mice

**Figure 4:**
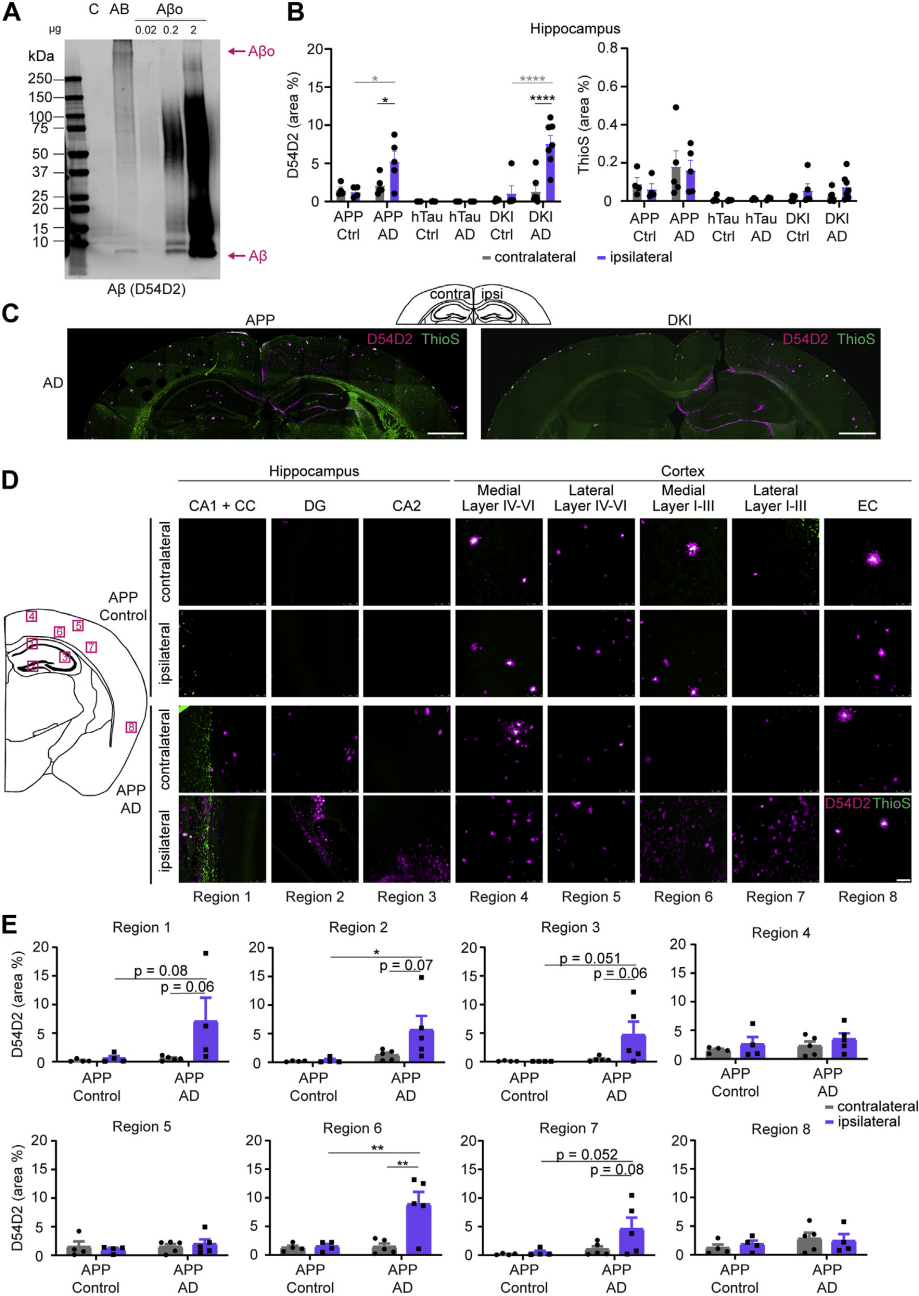
**Aβ in tau extracts leads to a redistribution of nondense core plaque Aβ on ipsilateral hemisphere.***A*, immunoblot of control (C) and brain A/B extract probed for Aβ with D54D2 antibody and compared with different amounts of synthetic biotinylated-Aβo. Samples were boiled for 5 min at 95 °C with 10% BME. *B*, quantification of the mean percent area occupied by D54D2-positive and ThioS-positive dense core plaques in the hippocampus of control and AD brain–injected mice was measured by drawing ROIs around the brain region and thresholding images. Representative images can be found in *C* and Fig. S3*A*. Statistics: Ordinary two-way ANOVA (for D54D2—interaction: *F*(5, 43) = 5.939, *p* = 0.0003; row factor: *F*(5, 43) = 12.25, *p* < 0.0001; column factor: *F*(1, 43) = 11.62, *p* = 0.0014; for ThioS—interaction: *F*(5, 43) = 0.4724, *p* = 0.7947; row factor: *F*(5, 43) = 5.546, *p* = 0.0005; column factor: *F*(1, 43) = 0.09148, *p* = 0.7638) with Sidak's multiple comparisons test comparing ipsilateral and contralateral hemisphere within each group shown in *black* and Sidak's multiple comparisons test comparing ipsilateral hemispheres between control and AD-injected mice shown in *gray*. N represents individual animals. N(APP-C) = 4, N(APP-AD) = 5, N(hT-C) = 4, N(hT-AD) = 3, N(DKI-C) = 4, and N(DKI-AD) = 7. ∗*p* < 0.05, ∗∗∗∗*p* < 0.0001. *C*, immunofluorescent staining with D54D2 (*magenta*) antibody for amyloid-β and thioflavin S (*green*) for dense-core amyloid-β plaques of APP and DKI mice injected with AD brain tau extracts (the scale bar represents 1 mm). *D*, representative images of D54D2 (*magenta*) and ThioS (*green*) staining taken with a 20× objective (the scale bar represents 50 μm). Schematic on the *left* indicates the location of images in the brain. *E*, quantification of D54D2 staining seen in *D*. For quantification of ThioS staining, see Fig. S3*B*. Statistics: Ordinary two-way ANOVA (for region 1—interaction: *F*(1, 13) = 2.449, *p* = 0.1416; row factor: *F*(1, 13) = 3.066, *p* = 0.1035; column factor: *F*(1, 13) = 3.131, *p* = 0.1002; For region 2—interaction: *F*(1, 14) = 2.309, *p* = 0.1509; row factor: *F*(1, 14) = 5.332, *p* = 0.0367; column factor: *F*(1, 14) = 2.655, *p* = 0.1255; region 3—interaction: *F*(1, 14) = 2.701, *p* = 0.1225; row factor: *F*(1, 14) = 3.560, *p* = 0.0801; column factor: *F*(1, 14) = 2.609, *p* = 0.1285; region 4—interaction: *F*(1, 14) = 0.005899, *p* = 0.9399; row factor: *F*(1, 14) = 0.8148, *p* = 0.3820; column factor: *F*(1, 14) = 1.500, *p* = 0.2410; region 5—interaction: *F*(1, 14) = 0.5541, *p* = 0.4690; row factor: *F*(1, 14) = 0.5692, *p* = 0.4631; column factor: *F*(1, 14) = 0.0001762, *p* = 0.9896; region 6—interaction: *F*(1, 14) = 7.892, *p* = 0.0139; row factor: *F*(1, 14) = 8.591, *p* = 0.0109; column factor: *F*(1, 14) = 8.843, *p* = 0.0100; region 7—interaction: *F*(1, 14) = 1.918, *p* = 0.1877; row factor: *F*(1, 14) = 4.515, *p* = 0.0519; column factor: *F*(1, 14) = 2.662, *p* = 0.1251; region 8—interaction: *F*(1, 14) = 0.3087, *p* = 0.5879; row factor: *F*(1, 14) = 1.470, *p* = 0.2470; column factor: *F*(1, 14) = 0.02451, *p* = 0.8780) with Sidak's multiple comparisons test comparing ipsilateral and contralateral hemisphere within each injection and ipsilateral results across injections. N represents individual animals. N(APP-C) = 4, N(APP-AD) = 4 and 5. ∗*p* < 0.05, ∗∗*p* < 0.01. AD, Alzheimer's disease; APP, amyloid precursor protein; DKI, double KI.

Immunoblot analysis of tau extracts generated from human AD patients revealed small amounts of residual monomeric Aβ as well as aggregated Aβo in the AD tau extracts (Fig. 4*A*). We explored whether the Aβ in the tau extracts contributed to tau spreading and whether it might alter Aβ deposition in APP and DKI mice. We stained brain sections of the tau-injected animals (Fig. 3) with an antibody recognizing the amino terminus of Aβ isoforms (D54D2) and thioflavin S (ThioS) to examine dense-core amyloid plaques. We then analyzed the percent area occupied by either stain in the whole hippocampus and cortex. When AD tau extracts containing Aβ are injected into mice, the ipsilateral hippocampus and parts of the overlying cortex exhibited a redistribution of Aβ along the hilus of the dentate gyrus, the outer edges of the dentate gyrus molecular layer, the hippocampal fissure, and the white matter surrounding the hippocampus and lower cortical regions (Fig. 4, *B* and *C*). This redistribution did not take place for Aβ that was part of dense-core plaques, since the ThioS signal was unaffected by AD extract injections (Fig. 4, *B* and *C*). Furthermore, redistribution was absent in control extract-injected animals (Fig. 4*B* and Fig. S3*A*). In animals without existing Aβ plaques (WT and hTau), injecting tau extracts containing Aβ was not sufficient to induce Aβ accumulation (data not shown). To further clarify the extent of Aβ redistribution, we imaged at higher magnification and measured the percent area occupied by total Aβ and ThioS-positive dense-core plaques in different regions of the hippocampus and cortex in APP mice. Most notably, redistribution of total Aβ took place in the CA1 and corpus callosum, dentate gyrus and CA2, as well as medial and lateral cortex layer I to III, whereas dense-core plaque signal remained unaltered in all regions except an overall signal increase in the corpus callosum (Fig. 4, *D* and *E* and Fig. S3*B*). Tile scanning of brain sections more posterior and anterior to the sections analyzed in Figure 4, *C*–*E* showed that the redistribution was present at a distance in and around the hippocampus far away from the injection site (Fig. S3*C*). Interestingly, comparing Aβ redistribution and the somatic and neuritic tau inclusions seen in these animals revealed very different patterns. Aβ redistribution was localized to the hippocampus and inner cortex layers and spread along the anterior–posterior axis but did not reach ventral or lateral brain regions (*e.g.*, EC). In comparison, somatic tau inclusions were present close to the injection site (hippocampus and RSA) and spread ventrally to the EC, potentially along synaptic connections.

**Figure 5:**
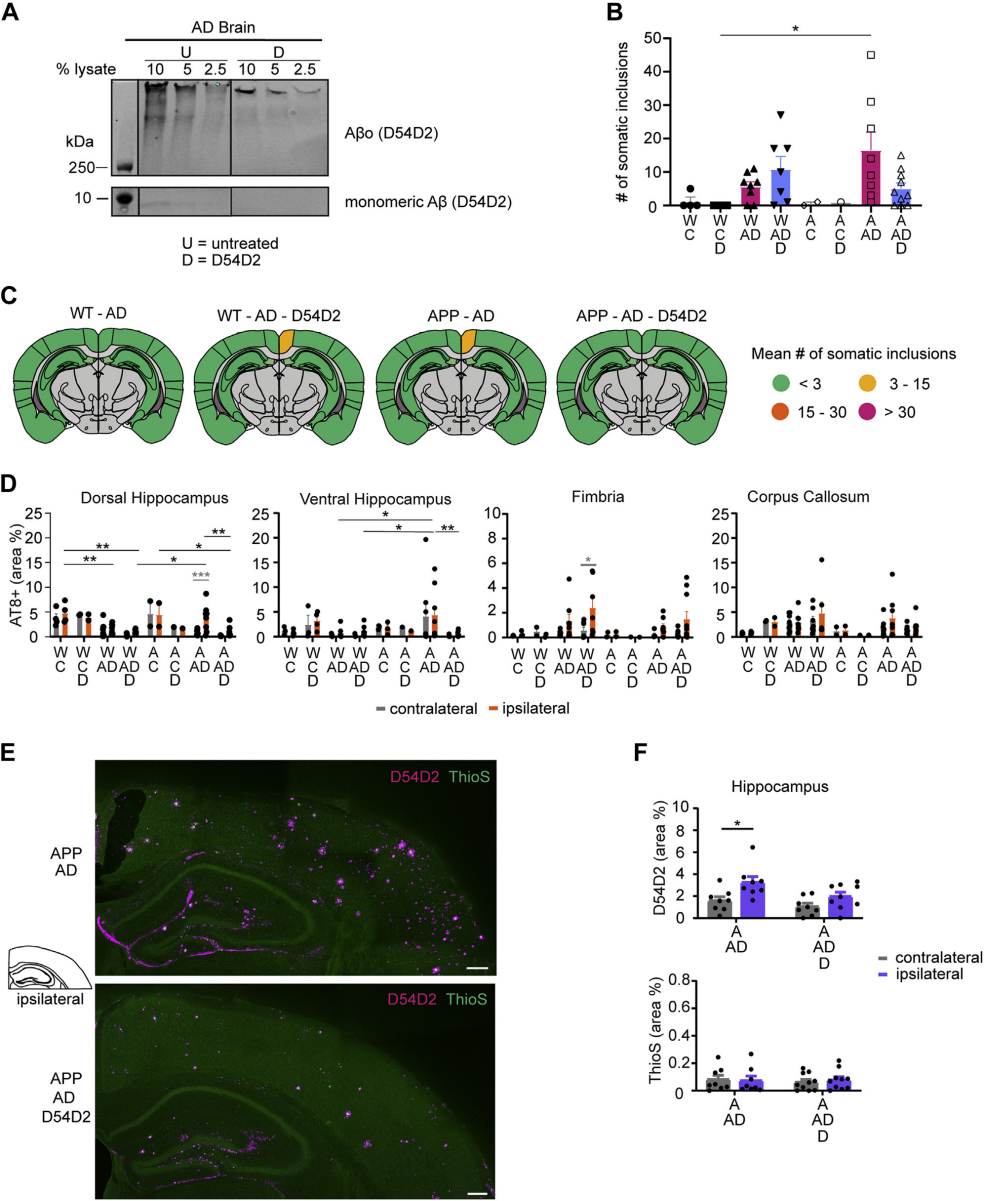
**Immunodepleting Aβ from tau extracts ameliorates Aβ redistribution.***A*, immunoblots of tau extracts that had been incubated with D54D2 antibody conjugated to Protein G magnetic beads to remove Aβ from tau extracts. Blots shown were probed for Aβ (D54D2) to assess the removal of Aβ from the extracts. Bands shown were run on the same blot but not next to each other, and *vertical black lines* indicate splicing of the blot. U = untreated samples, D = D54D2-incubated samples. *B*, mean number of somatic inclusions on the ipsilateral hemisphere of animals injected with control (C) or AD brain tau extracts, or with control or AD brain tau extracts that had been cleared of Aβ by incubating samples with D54D2 antibody conjugated to Protein G beads (C-D and AD-D samples). Statistics: Kruskal–Wallis test (approximate *p* value = 0.0176, Kruskal–Wallis statistic = 16.97) with Dunn's multiple comparisons test. N represents individual animals. N(WT-C) = 4, N(WT-C-D) = 4, N(WT-AD) = 8, N(WT-AD-D) = 7, N(APP-C) = 2, N(APP-C-D) = 1, N(APP-AD) = 8, and N(APP-AD-D) = 10. ∗*p* < 0.05. *C*, schematics of mean somatic inclusion burden of tau extract (extracted from AD brains, with or without Aβ removal)–injected animals dependent on mouse genotype. Brain regions not analyzed are depicted in *gray*. Ipsilateral hemisphere is on the *right*. *D*, area occupied by neuritic inclusions in four brain regions of animals injected with control or AD brain–derived tau extracts with and without Aβ removal. Statistics: Ordinary two-way ANOVA (for dorsal hippocampus—interaction: *F*(7, 68) = 1.680, *p* = 0.1287; row factor: *F*(7, 68) = 11.67, *p* < 0.0001; column factor: *F*(1, 68) = 1.790, *p* = 0.1864; for ventral hippocampus—interaction: *F*(7, 70) = 0.06833, *p* = 0.9995; row factor: *F*(7, 70) = 3.114, *p* = 0.0064; column factor: *F*(1, 70) = 0.07003, *p* = 0.7921; for fimbria—interaction: *F*(7, 61) = 0.7205, *p* = 0.6550; row factor: *F*(7, 61) = 1.275, *p* = 0.2777; column factor: *F*(1, 61) = 2.317, *p* = 0.1331; for corpus callosum—interaction: *F*(7, 67) = 0.2680, *p* = 0.9573; row factor: *F*(7, 67) = 1.825, *p* = 0.0967; column factor: *F*(1, 67) = 0.5514, *p* = 0.4603) with Sidak's multiple comparisons test. In *gray*: comparing ipsilateral and contralateral hemisphere for the same genotype and injected extract. In *black*: comparing ipsilateral values of different tau extract–injected groups. N(WT-C) = 4, N(WT-C-D) = 2, N(WT-AD) = 8, N(WT-AD-D) = 6 and 7, N(APP-C) = 2, N(APP-C-D) = 1, N(APP-AD) = 8, and N(APP-AD-D) = 10. ∗*p* < 0.05, ∗∗*p* < 0.01, ∗∗∗*p* < 0.005, and ∗∗∗∗*p* < 0.0001. *E*, representative images of the ipsilateral hippocampus with immunofluorescent staining of D54D2 (*magenta*) antibody for Aβ and ThioS (*green*) for dense-core amyloid plaques in APP mice injected with AD brain extracts with and without Aβ removal (the scale bars represent 250 μm). *F*, quantification of the percent area occupied by D54D2 (*top*) and ThioS (*bottom*) in the hippocampus of APP mice injected with AD brain extracts with and without Aβ removal. Statistics: Ordinary two-way ANOVA (for D54D2—interaction: *F*(1, 29) = 0.9156, *p* = 0.3465; row factor: *F*(1, 29) = 5.346, *p* = 0.0281; column factor: *F*(1, 29) = 10.96, *p* = 0.0025; for ThioS—interaction: *F*(1, 32) = 0.2028, *p* = 0.6555; row factor: *F*(1, 32) = 0.08193, *p* = 0.7765; column factor: *F*(1, 32) = 0.02327, *p* = 0.8797) with Sidak's multiple comparisons test comparing ipsilateral and contralateral hemisphere within each injection. N represents individual animals. N(APP-AD) = 8, N(APP-AD-D) = 8 to 10. ∗*p* < 0.05. AD, Alzheimer's disease; APP, amyloid precursor protein; DKI, double KI.

To discern whether the Aβ redistribution is dependent on the Aβ present in the tau extracts, we next performed immunodepletion of Aβ from tau extracts. We incubated tau extracts with magnetic beads conjugated to D54D2 (D) antibody and then proceeded to remove any additional D54D2 antibody from the extracts through an additional incubation with unconjugated beads. We immunoblotted untreated samples as well as Aβ-cleared extracts for both total Aβ and total tau (Fig. 5*A*). After immunodepletion, monomeric Aβ was absent from tau extracts. Oligomeric Aβ was reduced by 74% in Aβ-cleared tau extracts, but small amounts remained. Because of the nonspecific reduction of tau during Aβ immunodepletion, we adjusted the tau concentration of brain D extracts to match that of the Aβ D54D2-immunodepleted extracts for *in vivo* experiments focused on Aβ accumulation. With these tau-diluted samples, there was a lower somatic and neuritic inclusion burden on the ipsilateral and contralateral hemisphere of injected animals (Fig. 5, *B*–*D*).

We then examined whether the Aβ immunodepletion affects Aβ redistribution after injection of AD tau extracts into APP mice. Costaining with D54D2 antibody and ThioS showed that the redistribution of hippocampal Aβ accumulation was significantly reduced by the immunodepletion (Fig. 5, *E* and *F*). As was the case prior to immunodepletion, ThioS-stained area in APP mice was unaltered by human brain extract injection.

### Neither *Ptk2b* deletion nor treatment with the Fyn inhibitor AZD0530 altered tau spreading

**Figure 6:**
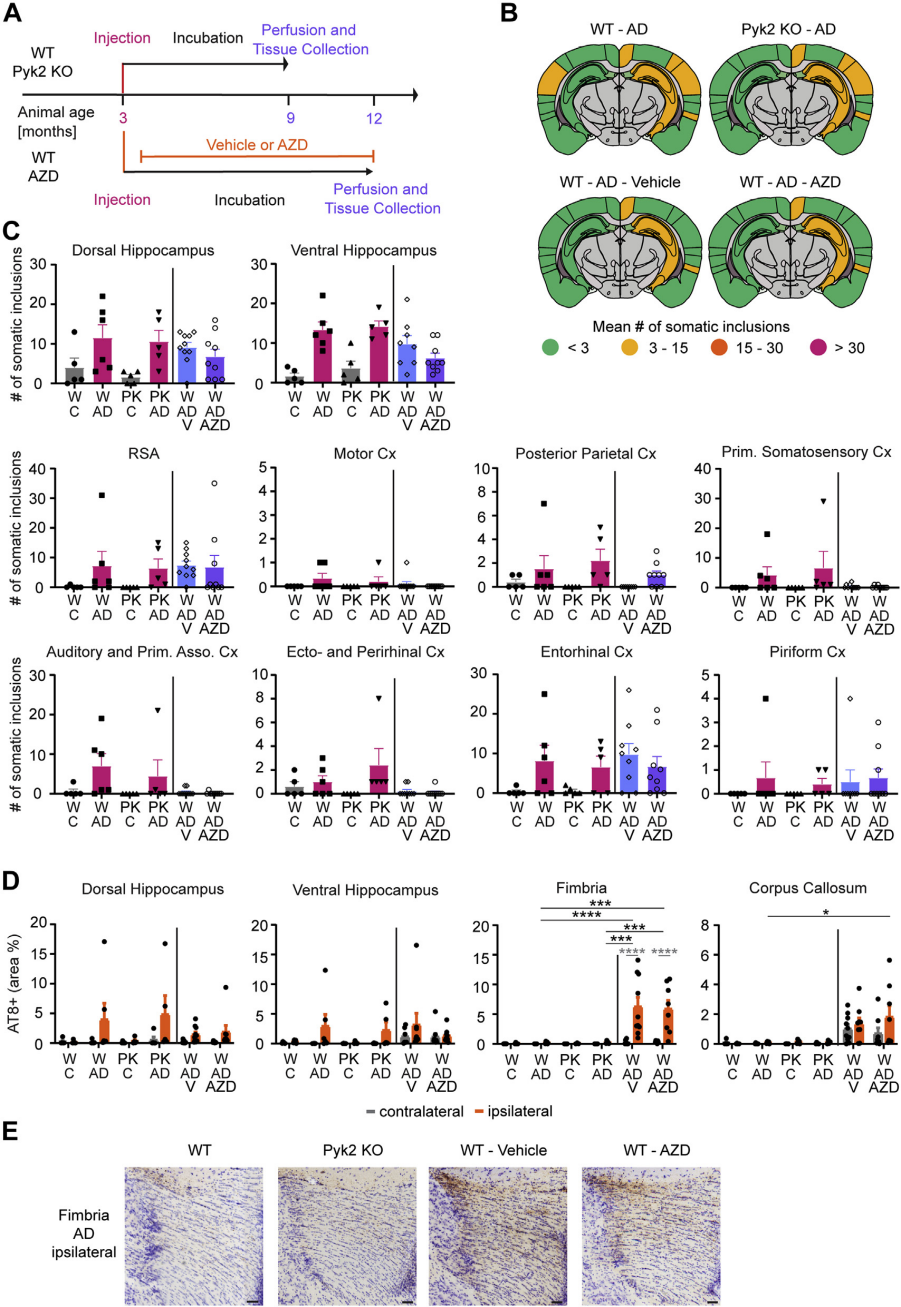
***Ptk2b***^***−/−***^**or pharmacological inhibition of Fyn have no impact on tau spreading.***A*, mice in the WT *versus Ptk2b*^*−/−*^ genotype comparison followed the established time line of injection at 3 months of age, waiting period of 6 months, and then tissue collection and analysis. WT animals treated with vehicle or AZD0530 were also injected at 3 months of age and then started on their AZD or vehicle treatment 2 weeks after tau injection. They were kept for 9 months before tissue was collected and analyzed. *B*, schematics of mean somatic inclusion burden of AD tau extract–injected animals dependent on mouse genotype. Brain regions not analyzed are depicted in *gray*. Ipsilateral hemisphere is on the *right*. *C*, mean number of somatic inclusions in ipsilateral brain regions of animals injected with tau extracts extracted from control or AD brains. C = control, W = WT, PK = *Ptk2b*^*−/−*^, V = vehicle treated, AZD = AZD0530 treated. Statistics: Kruskal–Wallis test (for dorsal HC—approximate *p* value = 0.563, Kruskal–Wallis statistic = 2.046; for ventral HC—approximate *p* value = 0.0153, Kruskal–Wallis statistic = 10.43; for RSA—approximate *p* value = 0.5301, Kruskal–Wallis statistic = 2.209; for motor cortex (Cx)—approximate *p* value = 0.3049, Kruskal–Wallis statistic = 3.625; for posterior parietal—approximate *p* value = 0.0217, Kruskal–Wallis statistic = 9.663; for primary somatosensory Cx—approximate *p* value = 0.0908, Kruskal–Wallis statistic = 6.472; for auditory Cx—approximate *p* value = 0.0209, Kruskal–Wallis statistic = 9.743; for ectorhinal and perirhinal CX—approximate *p* value = 0.0077, Kruskal–Wallis statistic = 11.9; for entorhinal Cx—approximate *p* value = 0.8443, Kruskal–Wallis statistic = 0.8217; for piriform Cx—approximate *p* value = 0.8054, Kruskal–Wallis statistic = 0.9828) with Dunn's multiple comparisons test. N represents individual animals. N(WT-C) = 5, N(WT-AD) = 6, N(PK-C) = 5, N(PK-AD) = 5, N(WT-AD-V) = 8 to 10, and N(WT-AD-AZD) = 9. ∗*p* < 0.05, ∗∗*p* < 0.01. *D*, area occupied by neuritic inclusions in four regions of animals injected with tau extracts. Statistics: Ordinary two-way ANOVA (for dorsal hippocampus—interaction: *F*(5, 66) = 1.122, *p* = 0.3574; row factor: *F*(5, 66) = 1.341, *p* = 0.2580; column factor: *F*(1, 66) = 8.063, *p* = 0.0060; for ventral hippocampus—interaction: *F*(5, 63) = 0.7387, *p* = 0.5973; row factor: *F*(5, 63) = 1.155, *p* = 0.3411; column factor: *F*(1, 63) = 4.363, *p* = 0.0408; for fimbria—interaction: *F*(5, 62) = 5.641, *p* = 0.0002; row factor: *F*(5, 62) = 6.684, *p* < 0.0001; column factor: *F*(1, 62) = 12.88, *p* = 0.0007; for corpus callosum—interaction: *F*(5, 63) = 0.7336, *p* = 0.6010; row factor: *F*(5, 63) = 5.642, *p* = 0.0002; column factor: *F*(1, 63) = 1.505, *p* = 0.2244) with Sidak's multiple comparisons test. In *gray*: comparing ipsilateral and contralateral hemisphere of the same genotype and injected extract. In *black*: comparing ipsilateral values of different AD tau extract–injected groups. N represents individual animals. N(WT-C) = 5, N(WT-AD) = 6, N(PK-C) = 5, N(PK-AD) = 5, N(WT-AD-V) = 8 to 10, and N(WT-AD-AZD) = 8 and 9. ∗*p* < 0.05, ∗∗*p* < 0.01. *E*, representative images of neuritic inclusions in the fimbria (the scale bar represents 50 μm). AD, Alzheimer's disease; APP, amyloid precursor protein; DKI, double KI; RSA, retrosplenial area.

Pyk2 was demonstrated to be a tau kinase (67), and Fyn kinase inhibition or deletion reduced Tau deposition and rescued memory deficits in several tauopathy models (68, 73, 74). Therefore, we sought to determine whether inhibiting Fyn kinase or knocking out Pyk2 would modify tau spreading. Mice were injected with AD tau extracts at 3 months of age, and Fyn kinase inhibitor treatment with AZD0530 (AZD) was started 2 weeks after the injection. WT and *Ptk2b*^*−/−*^ brain tissue was collected 6 months after the injection, whereas vehicle *versus* AZD-treated WT animals were maintained until 9 months after injection (Fig. 6*A*). Overall, there were no significant changes in the number of somatic inclusions on the ipsilateral and contralateral hemispheres of injected animals when comparing WT to *Ptk2b*^*−/−*^ animals and vehicle-dosed animals to AZD-treated animals (Fig. 6, *B* and *C*). Interestingly, the three additional months of incubation time granted to the vehicle- and AZD-treated animals resulted in an increase of neuritic inclusions in the fimbria and corpus callosum independent of drug or vehicle treatment (Fig. 6, *D* and *E*).

### Deficiency of PGRN or TMEM106B does not alter tau accumulation, but aging enhances tau spreading and inclusion burden

As PGRN and TMEM106B have both been reported to regulate lysosomal biology (28, 75), we sought to evaluate whether PGRN or TMEM106B deficiency would modulate tau spreading. Here, we also considered whether advanced age would interact with the *Tmem106b* genotype since aging is a key risk factor for AD. Mice homozygous for the hypomorphic *Tmem106b* allele show some lysosomal abnormalities but have no observable alteration in life span facilitating these studies (76).

**Figure 7:**
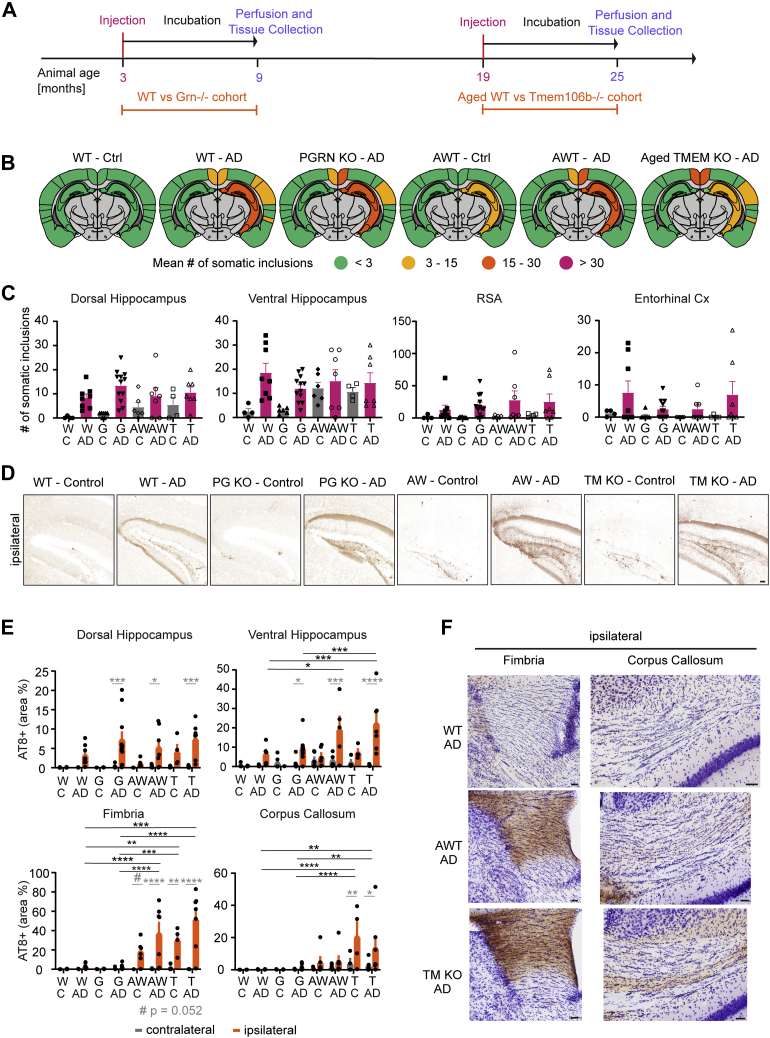
***Grn***^***−/−***^**and *Tmem106b***^***−/−***^**do not impact tau spreading, but advanced mice age exacerbates contralateral hippocampal inclusions and neuritic tau deposition.***A*, WT and *Grn*^*−/−*^ mice were injected according to the time line previously described. Aged WT and *Tmem106b*^*−/−*^ mice were injected at 19 months of age instead of 3 months of age and then followed the same time line of 6-month waiting period, followed by mice perfusion, tissue collection, and analysis. *B*, schematics of mean somatic inclusion burden of control and AD tau extract–injected animals dependent on mouse genotype and age at injection. Brain regions not analyzed are depicted in *gray*. Ipsilateral hemisphere is on the *right*. *C*, mean number of somatic inclusions in ipsilateral brain regions of animals injected with tau extracts from control or AD brains. C = control, W = WT, G = *Grn*^*−/−*^, AW = aged WT, and T = *Tmem106b*^*−/−*^. Statistics: Kruskal–Wallis test (for dorsal HC—approximate *p* value = 0.3227, Kruskal–Wallis statistic = 3.485; for ventral HC—approximate *p* value = 0.5686, Kruskal–Wallis statistic = 2.018; for RSA—approximate *p* value = 0.6358, Kruskal–Wallis statistic = 1.705; for entorhinal Cx—approximate *p* value = 0.9075, Kruskal–Wallis statistic = 0.5511) with Dunn's multiple comparisons test comparing AD-injected groups. N represents individual animals. N(WT-C) = 4, N(WT-AD) = 8, N(G-C) = 7, N(G-AD) = 12, N(AWT-C) = 6, N(AWT-AD) = 7, N(T-C) = 4, and N(T-AD) = 7. *D*, representative images of ventral hippocampi of animals injected with control or AD tau extracts (the scale bar represents 50 μm). *E*, area occupied by neuritic inclusions in four brain regions of animals injected with control or AD brain–derived tau extracts. Statistics: Ordinary two-way ANOVA (for dorsal hippocampus—interaction: *F*(7, 91) = 3.335, *p* = 0.0033; row factor: *F*(7, 91) = 3.823, *p* = 0.0011; column factor: *F*(1, 91) = 32.08, *p* < 0.0001; for ventral hippocampus—interaction: *F*(7, 83) = 5.547, *p* < 0.0001; row factor: *F*(7, 83) = 5.329, *p* < 0.0001; column factor: *F*(1, 83) = 32.05, *p* < 0.0001; for fimbria—interaction: *F*(7, 94) = 10.82, *p* < 0.0001; row factor: *F*(7, 94) = 11.02, *p* < 0.0001; column factor: *F*(1, 94) = 59.22, *p* < 0.0001; for corpus callosum—interaction: *F*(7, 94) = 2.355, *p* = 0.0292; row factor: *F*(7, 94) = 5.079, *p* < 0.0001; column factor: *F*(1, 94) = 12.36, *p* = 0.0007) with Sidak's multiple comparisons test. In *gray*: comparing ipsilateral and contralateral hemisphere for the same injected extract and genotype. In *black*: comparing ipsilateral values of AD tau extract–injected groups. N represents individual animals. N(WT-C) = 4, N(WT-AD) = 8, N(G-C) = 7, N(G-AD) = 12, N(AWT-C) = 6, N(AWT-AD) = 7, N(T-C) = 4, and N(T-AD) = 7. ∗*p* < 0.05, ∗∗*p* < 0.01, ∗∗∗*p* < 0.005, and ∗∗∗∗*p* < 0.0001. *F*, representative images of fimbria and corpus callosum neuritic tau inclusions (the scale bars represent 50 μm). AD, Alzheimer's disease; RSA, retrosplenial area.

We injected WT (W) and *Grn*^*−/−*^ (G) mice with control or AD patient–derived tau at 3 months of age, whereas aged WT (AW) and *Tmem106b*^*−/−*^ (T) mice were 19 months old at the time of injection and 25 months at the end of the experiment (Fig. 7*A*). AD extract–injected animals showed comparable amounts of somatic inclusions on both hemispheres, regardless of animal age and genotype (Fig. 7, *B* and *C*). Interestingly, the control extract injection induced somatic tau inclusions in aged mice but not in young mice. This phenomenon depended on mouse age, not genotype, and was spatially restricted close to the injection site (Fig. 7*D*). Furthermore, advanced mouse age also had an impact on the density of neuritic tau inclusions, causing significant increases in the ventral hippocampus, fimbria, and corpus callosum in AD extract–injected animals (Fig. 7, *E* and *F*). As for somatic tau inclusions, mouse genotype did not alter the neuritic tau inclusion burden. These findings expand evidence that aging renders cells more susceptible to tau seeding and spreading. In contrast, disrupting lysosomal degradation by knocking out either PGRN or TMEM106B had no impact on tau spreading.

## Discussion

In the present study, we examined cellular and molecular processes that might modify tau spreading in the context of AD. We extracted tau seeds from human AD subject brains and injected them into WT and transgenic mice as described previously (11, 18, 41). We observed that Aβ pathology, Fyn or Pyk2 inhibition, and lysosomal dysregulation by PGRN or TMEM106B deficiency have no significant effects on tau spreading, while animal age and the presence of hTau promoted the deposition of neuritic, but not somatic, tau.

We characterized the quality and seeding capabilities of our tau extracts. It appears that the extracts here contain tau isoforms similar to those in the literature (18) and also contain low levels of Aβ. The *in vitro* seeding capabilities of our extracts also match previous findings (18). As the tau/total protein ratio in our extracts ranged between 1.6 and 9.5% tau content by immunoblot, we injected 0.5 μg/site with brain AB extracts, whereas other groups used 1 to 4 μg/site (11, 18, 41, 77). While dose–response relationships were ill defined, the lower concentrations may explain the limited somatic tau inclusions in WT animals, and why only regions with the highest amounts of tau deposition were significantly different from control-injected animals. However, the pattern of hippocampal and cortical regions with highest accumulation was consistent with previous studies (18).

There has been a longstanding debate regarding how Aβ-induced signaling and/or Aβ plaques themselves facilitate or even initiate the formation of tau deposits. On one hand, in mouse models overexpressing both mutant APP and tau, NFT formation is accelerated by the presence of Aβ (44, 45). On the other hand, for models dealing with injected tau or Aβ, the evidence is less clear. Injecting artificial Aβ_42_ fibrils into a mouse model overexpressing P301L mutant tau led to a fivefold increase in NFTs (42). In contrast, studies using a human tau injection model reported a decrease in the number of NFT in the hippocampus and EC in 5xFAD mice, accompanied by an increase in NP tau instead (41, 78). In this model, microglial ablation and TREM2 knockout further enhanced NP tau deposition (78). Injecting the less severe *App*^*NL-F/NL-F*^ mouse model did not alter NFT or NP tau burden (41). In a fourth study, hTau mice were crossed with *App*^*NL-G-F/NL-G-F*^ mice (carrying the Arctic mutation in addition to the Swedish and Iberian mutations) and injected with tau seeds (72). That study observed an increase in tau deposition for mice carrying hTau in comparison to WT animals, and this increase was further exacerbated in mice carrying both hTau and *App*^*NL-G-F/NL-G-F*^ mutations.

Here, we observed equally increased somatic inclusions in AD tau extract–injected hTau or hTau-*App*^*NL-F/NL-F*^ (DKI) mice as compared with WT animals. Both groups had greater tau inclusions than mice with murine tau, and this aligns with evidence that the presence of hTau in mice increases tau deposition after tau seed injection (72), but that the *App*^*NL-F/NL-F*^ genotype had no impact on tau spreading (41). When analyzing another Aβ-overexpression mouse model (*APPswe/PSEN1ΔE9*, termed APP here), we expected to see a decrease in NFT and increase in NP tau similar to that seen in 5xFAD mice, but we did not observe any changes in somatic inclusion burden. In addition, we detected no increase in NP tau in AD extract–injected DKI or APP animals. We hypothesize that this might be due to the greatly accelerated Aβ pathology in 5xFAD compared with APP or DKI mice, with 5xFAD mice showing higher plaque burden at a younger age (79). Potentially, this provides an earlier and more aggressive microenvironment around plaques that is conducive to the aggregation of NP tau. Thus, we speculate that injecting APP/PS1 mice at 9 months instead of 3 months of age might lead to a phenotype similar to the one observed in 5xFAD mice. Another observation supporting the hypothesis that only high levels of Aβ burden are able to impact tau accumulation is that hTau-*App*^*NL-G-F/NL-G-F*^ mice injected with tau show increased tau deposition compared with hTau mice (72), while we observed no exacerbation in mice with the less severe Aβ phenotype, hTau-*App*^*NL-F/NL-F*^ mice. While Aβ pathology at the levels of *APPswe/PSEN1ΔE9* or *App*^*NL-F/NL-F*^ mice did not alter tau spreading from injected mature AD seeds, this does not address the issue of whether Aβ-dependent mechanisms initiate tau misfolding separately from the spread of preformed seeds.

The hypothesis of Aβ-independent tau spreading with moderate levels of Aβ accumulation is further supported by the fact that Aβ and tau show distinct spreading patterns in both human and animal models (19, 20, 21, 47). We observed a redistribution of Aβ accumulation on the ipsilateral hemisphere, whereas dense-core plaques and tau depositions remained unaffected by the injection. The redistribution was not induced by injecting control extract and was ameliorated by partial immunodepletion of Aβ. Thus, we conclude that the redistribution was dependent on the small amounts of residual Aβ in our tau extracts. In support of this notion, the pattern of redistribution we observed is similar to the deposition reported by other groups (80, 81, 82) when injecting Aβ extracted from human brains into mice.

Fyn and Pyk2 are tyrosine kinases that have been reported to directly interact with and phosphorylate tau (65, 66, 67, 68) and to also increase the activity of glycogen synthase kinase 3β (69, 70, 71). A recent study has shown increases in tau phosphorylation at Y18 in human Pyk2/P301L tau double transgenic mice (67). Other studies found that Fyn also phosphorylated tau at Y18 (65) and that tau recruits Fyn to synapses (66). Furthermore, knocking out or pharmacologically inhibiting Fyn in human tauopathy models led to a decrease in NFT, tau phosphorylation, and synaptic tau accumulation (68, 73, 74). We thus expected that inhibiting either one of these kinases in WT mice, by knocking out Pyk2 or pharmacologically inhibiting Fyn, might decrease tau phosphorylation, resulting in reduced tau deposition after tau seed injection. However, we observed no effect of kinase inhibition or deletion on tau spreading. There are several possible explanations for the negative results. The first one is that other kinases can compensate for the loss of one kinase. This would imply that we would have to disrupt several kinases at the same time to achieve an observable effect on tau spreading by AD tau seeds. Another one is that the phospho-tau epitopes targeted by Fyn and Pyk2 are important for the initial *de novo* tau misfolding and aggregation and but are not critical after the initial seeding event has taken place, and templating of conformation is key. By injecting exogenous “mature” tau seeds, the current study focuses exclusively on templating and spreading of tau but does not assess the initial *de novo* seed formation steps in tau pathology. There is also evidence in the literature supporting this hypothesis since inhibiting Fyn in WT animals immediately after tau pathology was induced by traumatic brain injury–reduced phospho-tau accumulation and synapse loss, while initiating Fyn inhibition 100 days after the injury showed no effect (73).

Another cellular process dysregulated in AD is autophagy (24, 25). We focused on two proteins (TMEM106B and PGRN) that regulate lysosomal function. Reduction of TMEM106B by hypomorphic *Tmem106b* alleles was reported to decrease levels of lysosomal enzymes and to disrupt lysosomal acidification, one of the final steps in autophagy, leading to impaired degradation (32). PGRN deficiency was reported to cause increases in lipofuscin and in the levels of many lysosomal enzymes (32). Furthermore, decreasing PGRN levels were reported to enhance tau phosphorylation in P301L mutant tau mice (35, 36). However, the mechanistic details of this enhanced tau phosphorylation remain elusive. Given the previously reported effects of TMEM106B and PGRN deficiency, we hypothesized an increase in tau spreading in the two knockout mice. Surprisingly, we observed no significant effects on tau spreading when disrupting expression of either TMEM106B or PGRN. The lack of effect on tau spreading might be due to compensation by other cellular mechanisms (*e.g.*, upregulated ubiquitin–proteasome response). Alternatively, as for the synaptic tyrosine kinases, lysosomal degradation may be more relevant for clearing *de novo* seed formation than for modulating tau templating and propagation.

Tau spreading was strongly increased by the presence of human tau and by advanced mouse age. We were able to confirm that the presence of hTau increases neuritic tau deposition in mice. Previous studies reported that tau from a particular species might template best onto tau of that same species (72, 77). This matches with the idea that a substrate with an amino acid match to the seed is more energetically favored to match the misfolded state of the seed.

The effect of age on tau spreading is most interesting, and an increase in somatic and neuritic tau seeding has been detected in older mice previously (18). In humans, aging is one of the most important risk factors for developing sporadic AD, and it is suspected that a variety of factors, including impaired protein clearance mechanisms, contribute to susceptibility and development of neurodegenerative diseases (83). Our data indicate that tau deposition is increased when injecting tau seeds into older animals but disrupting lysosome-mediated clearance in aged mice by knocking out TMEM106B did not further increase tau deposition. Recently, heparin-independent aggregation of synthetic tau was shown to generate fibrils that are similar to brain-extracted tau fibrils (84). Evaluating their seeding potential in mice models would be important, since we did observe differences in seeding density between extracts of different human brains, and these fibrils could be a useful tool to standardize future tau injection studies. Furthermore, it will be of interest to dissect which aging mechanisms impact initial tau seed formation *versus* tau spreading *versus* both. Identifying those mechanisms that are necessary and sufficient to generate *de novo* tau seeds may be most relevant for the development of early intervention treatments for AD. Conversely, understanding of how tau spreading is regulated could pave the way for treatments of later stages of this devastating disease, when first tau deposition has taken place, but pathology has not spread throughout the brain yet.

## Experimental procedures

### Animals

C57BL/6J mice (RRID: IMSR_JAX: 000664) for cohorts injected with brain A, B, AB, D, brain D_conc_ or treated with vehicle or AZD were purchased from Jackson Laboratories (JAX) and arrived at 8 to 9 weeks of age. *APPswe/PSEN1ΔE9* mice on a C57BL/6J background had been purchased from JAX (RRID: MMRRC_034832-JAX; Jankowsky *et al.* (85)) and maintained in our institution's animal facility. *Ptk2b*^*−/−*^ mice (RRID: MGI:3584536; Okigaki *et al.* (86)) on the C57BL6J background after ten backcrosses were generously provided by Dr David Schlaepfer (University of California, San Diego) and maintained in our institution's animal facility. *Tmem106b*^*−/−*^ mice on the C57BL/6N background were generated previously by a lacZ gene trap strategy (32). The *Tmem106b*^*−/−*^ gene trap line is a hypomorph and expresses 5 to 10% residual full-length TMEM106B protein (76, 87). *Grn*^*−/−*^ mice on a C57BL/6J background (RBRC02370; Kayasuga *et al.* (88)) were obtained from RIKEN BioResource Center and bred at our institution's animal facility. *App*^*NL-F/WT*^ heterozygous KI mice were imported from the RIKEN Institute. In these mice, one allele of the APP gene contains three point mutations to humanize the Aβ sequence and also the Swedish (KM670/671NL) and Iberian (I716F) mutations (89). The mice were backcrossed for more than ten generations to C57BL/6J strain and then expanded to generate homozygous *App*^*NL-F/NL-F*^ mice (NLF) and *App*^*WT/WT*^ WT controls. Subsequently, these homozygous mice were crossed with *B6.Cg-Mapt<tm1.1(MAPT)Tcs>* (from now on called *hTau*) KI mice (RBRC09995; Saito *et al.* (72)) obtained from RIKEN BioResource Center to generate *hTau-App*^*NL-F/NL-F*^ mice (DKI).

All experiments with mice bred at our institution's animal facility used littermate control mice (C57BL/6J background) with no preference for male or female mice. See Fig. S1*C* for details on mice used for each injection cohort. All animals were cared for by our institutions Animal Resource Center until the time of injection (at around 12 weeks of age unless indicated otherwise) and returned there until they were perfused, and tissue was collected. All protocols were approved by our Institutional Animal Care and Use Committee. The animals were housed in groups of 2 to 4 animals per cage with *ad libitum* access to food and water. The housing light has a scheduled light period from 7 AM to 7 PM and a dark period for the remaining 12 h.

### Chronic oral dose preparation of AZD

*N*-(5-chloro-1,3-benzodioxol-4-yl)-7-[2-(4-methylpiperazin-1-yl)ethoxy]-5-(tetra-hydro-2H-pyran-4-yloxy)quinazolin-4-amine (AZD; saracatinib) was prepared as described previously (18, 73). Mice received a drug dosage of 5 mg/kg per day through purified diet pellets. The drug dosage in the food was calculated to take into account the average amount of food eaten by a mouse in a single day per kilogram of weight (90). The compound was incorporated into purified diet pellets by Research Diets, Inc, by dissolving the compound in a solution of 0.5% w/v hydroxypropylmethylcellulose/0.1% w/v polysorbate 80 at 1.429 mg/ml to dose animals chronically. For vehicle pellets, diet pellets were purified with control vehicle solution (without drug).

### Tau extraction

Pre-existing deidentified human autopsy brains were accessed for these studies under conditions considered exempt from Human Subjects regulations after review of our Institutional Review Board. Fresh-frozen brain had been stored at −80 °C; see Fig. S1*A* for post-mortem information on the brains. Tau was extracted based on a previously published protocol (18) with some modifications. Briefly, 11 to 12 g of cortical gray matter were dounce homogenized in 30 ml lysis buffer (10 mM Tris–HCl, 1 mM EDTA, 0.1% sarkosyl, 10% sucrose, freshly added 2 mM DTT, phosSTOP [Roche], and protease inhibitors [Roche]). During the extraction, lysates were kept on ice. Homogenates were centrifuged at 12,000 rpm at 4 °C for 12 min (Ti 45 rotor; Beckman Coulter). The supernatant was pooled, and the pellets were re-extracted and centrifuged twice more as aforementioned. The pooled supernatant was centrifuged twice more at 12,000 rpm at 4 °C for 12 min (Ti 45 rotor; Beckman) to remove debris. Then, the sarkosyl concentration was increased to 1%, and samples were nutated for 1 h at room temperature (RT). The samples were centrifuged at 300,000*g* for 1 h at 4 °C (57,000 rpm, Ti 70 rotor; Beckman Coulter). The resulting pellet was washed with PBS supplemented with phosSTOP and protease inhibitors twice and then resuspended in PBS supplemented with phosSTOP and protease inhibitors. After sonication at 15% amplitude for 20 s with 0.5 s ON/0.5 s OFF pattern, and the lysate was centrifuged at 100,000*g* for 30 min at 4 °C. The supernatant was discarded, and the pellet washed twice in PBS supplemented with phosSTOP and protease inhibitors. The pellet was once more resuspended in PBS supplemented with phosSTOP and protease inhibitors and sonicated at 30% amplitude for 60 s with 0.5 s ON/0.5 s OFF pattern. This was followed by a 100,000*g* spin for 30 min at 4 °C. The resulting supernatant contained the soluble tau and was aliquoted and stored at −80 °C until further analysis or experimental use. Tau extract concentration was determined by comparing tau extracts diluted to 10%, 5%, or 2.5% in 1× Laemmli sample buffer to the same dilution curve of recombinant 2N4R tau (#842501; Biolegend). Total protein concentration in tau extracts was assessed by measuring absorption at 280 nm on a Nanodrop Spectrophotometer (#N-1000; Thermo Fisher Scientific).

### Atomic force microscopy

Samples were prepared by splitting Mica discs (#50, 9.9-mm diameter; Ted Pella, Inc, PELCO Mica Discs) with a fresh razor and sticking them to a glass coverslip with a double-sided sticky tab (#16084-6, 6 mm OD; Ted Pella, Inc, PELCO Tabs). Tau extracts were diluted to a tau concentration of 5 ng/μl, and mica discs were covered with 10 μl of tau extract for 2 min. Afterward, the discs were washed twice with 100 μl double-distilled water (ddH_2_O) and stored protected from light at RT until imaging. Samples were scanned at a Dimension FastScan with ScanAsyst AFM (Bruker) with a Fastscan-B (Bruker) cantilever in ScanAsyst air mode. Scans were 5 × 5 μm in size, with a scan rate of 3.38 Hz and 1024 samples/line. After acquisition, images were processed with Research NanoScope software (Bruker) by flattening images and adjusting the z-scale from −10 to 20 nm. Tau fibril length was manually analyzed by measuring the distance between two points in Gwyddion (GPL, free software). For each tau extract, 2 to 3 images were analyzed with 50 to 200 fibrils measured per image.

### *In vitro* tau seeding in mouse primary neurons

Primary mouse neuronal culture was prepared as described previously (32). Pregnant mice were euthanized with CO_2_, and hippocampal and cortical tissues (1:1 ratio) were harvested from E17 embryos on ice-cold Hibernate E media (BrainBits, HE), digested in 0.05% trypsin (Gibco) and 1 mg/ml DNase (Sigma DN25) in Hanks' balanced salt solution for 10 min at 37 °C. After incubation, neurons were triturated manually in neurobasal-A media (Gibco) supplemented with B27 (Gibco), 1 mM sodium pyruvate, GlutaMAX (Gibco), 100 U/ml penicillin (Gibco), and 100 μl streptomycin (Gibco) at 37 °C. Dissociated neurons were spun at 250*g* at 4 °C for 6 min and plated at 50,000 to 75,000 cells/well onto PDL-coated 96-well plates (Corning; #354461) in the same neurobasal-A media with supplements as aforementioned. *In vitro* tau seeding experiments were performed as previously described (73) with modifications. On DIV 7, tau extracts 0.25% (v/v) from human AD brains were seeded into wells. At DIV 21, neurons were fixed with ice-cold methanol for 30 min on ice and blocked with 10% normal donkey serum and 0.2% Triton X-100 in PBS for 30 min. Then, neurons were incubated with primary antibodies diluted in 1% normal donkey serum and 0.2% Triton X-100 in PBS overnight at 4 °C: Anti-MAP 2 (Cell Signaling; #4542, 1:150) and mouse Tau (T49) (MilliporeSigma; #MABN827, 1:500). The samples were washed three times with PBS and incubated in secondary antibodies (Invitrogen Alexa Fluor 1:500) and 0.5 μg/ml 4′,6-diamidino-2-phenylindole diluted in 1% normal donkey serum and 0.2% Triton X-100 in PBS for 1 h.

### Stereotactic surgery on mice

Mice were injected as previously described (18). For detailed information on animal numbers in injected mouse cohorts, see Fig. S1*C*.

Briefly, mice received 0.05 mg/kg buprenorphine (Buprenex injection, 0.03 mg/ml; Reckitt Benckiser Healthcare Ltd, diluted 1:10 in PBS before use) 30 min before undergoing surgery. They were anesthetized by placing them in an isoflurane (Covetrus) and oxygen filled chamber and kept in anesthesia with 2 to 3% isofluorane mixed with oxygen (Quantiflex Low Flow V.M.C.; Matrix Medical Inc). Animals were immobilized in a stereotaxic frame (David Kopf Instruments), and their skull was shaved, followed by disinfecting the incision site three times with 70% ethanol and iodine. An incision of approximately 1-cm length was made on the animals' skull. Extracted human tau extracts were aseptically injected using a Hamilton syringe (#901; Hamilton) with a 33-gauge needle, 45° tip (#7803-05; Hamilton), controlled by a Micro4 Microsyringe Pump Controller (World Precision Instruments) at a rate of 0.25 μl/min under stereotactic guidance at two locations. The first location was in the hippocampus, the other in the overlying cortex (from bregma: anterior–posterior −2.5 mm; medial–lateral 2 mm; dorsoventral −2.4 mm [for hippocampus], and dorsoventral −1.4 mm [for cortex]). Each location received 2.5 μl of human tau. The hippocampus was injected first, and after each injection, there was a waiting period of 3 min to allow the injected solution to permeate into the tissue. After injections were completed, animals were closed with 2 to 3 surgical sutures (Synthetic Absorbable Vicryl Suture, #J310; Ethicon) and monitored until they regained responsiveness.

For postoperative pain management, animals received 0.05 mg/kg buprenorphine for 3 days (twice daily, 12 h apart) as analgesic, accumulating to a total of six buprenorphine injections per animal. The incision was checked daily for infections and/or pulled stitches, and if necessary, sutures were replaced.

### Mouse brain tissue collection and processing

Six (or nine) months after injection, mice were anesthetized with CO_2_ for 45 s, followed by transcardial perfusion with 20 ml of ice-cold PBS. Brains were extracted and postfixed for 48 h in 4% paraformaldehyde. Afterward, brains were stored in PBS containing 0.05% NaN_3_ and sectioned with a vibratome into 40-μm thick coronal sections (Leica VT1000S). To be able to identify ipsilateral and contralateral hemispheres relative to the tau injection after immunohistochemistry (IHC), the contralateral hemisphere received a small incision in the auditory cortex before sectioning.

### IHC

#### Fluorescent staining

Unless indicated otherwise, free-floating sections were washed once in blocking buffer (1% bovine serum albumin + 1% Triton-X in PBS) for 5 min, followed by incubation in blocking buffer for 1 h at RT. Sections were then incubated with primary antibodies diluted in blocking buffer for 48 to 72 h at 4 °C. Afterward, sections were washed three times for 5 min in blocking buffer or PBS and incubated overnight at 4 °C with the appropriate secondary antibodies (AlexaFluor 488, 568, or 647, diluted 1:500 in blocking buffer). The next morning, sections were incubated three times for 5 min with PBS, followed by copper sulfate treatment to reduce autofluorescence. For copper sulfate treatment, sections were briefly transferred to ddH_2_O, incubated for 15 min in copper sulfate solution (10 mM CuSO_4_, 50 mM ammonium acetate, and pH 5), briefly returned to ddH_2_O, and incubated in PBS for at least 10 min. Afterward, sections were mounted on microscope slides (#22-178-277; Fisher Scientific) and coverslipped with VECTASHIELD Antifade Mounting Medium containing 4′,6-diamidino-2-phenylindole (#H-1200; Vector Laboratories). See Table 1 for details on antibody information.

**Table 1:** Primary antibodies and dyes used in this work

Name	Catalog number	Manufacturer	Host species	Dilution	Application
Aβ (D54D2)	8243S	Cell Signaling Technologies	Rabbit	1:500	IHC
p-Tau (Thr231) AT180	MN1040	Invitrogen	Mouse	1:1000	WB
Biotinylated p-Tau (Ser202/Thr205) AT8-B	MN1020B	Invitrogen	Mouse	1:250	DAB
CD68 (FA-11)	MA5-16674	Invitrogen	Rat	1:250	IHC
Cresyl violet	C5042-10G	Sigma–Aldrich	—	1 g/l	Nissl stain
GFAP	Z0334	DAKO	Rabbit	1:250	IHC
MAP2	4542	Cell Signaling Technologies	Rabbit	1:150	IHC
Mouse tau (T49)	MABN827	MilliporeSigma	Mouse	1:500	IHC
ThioS	T1892-25G	Sigma–Aldrich	—	0.1% (w/v) in 70% ethanol	ThioS
Total tau	MN1000	Invitrogen	Mouse	1:1000	WB

For costaining of total Aβ and dense core Aβ plaques, ThioS staining was performed after sections had been stained for Aβ. Sections were incubated for 15 min in 0.1% ThioS in 70% ethanol, followed by two 5-min washes in 70% ethanol, and two 5-min washes in ddH_2_O. Sections were then returned to PBS and mounted as described before.

#### 3,3'-Diaminobenzidine IHC and Nissl stain

To perform 3,3'-diaminobenzidine (DAB) IHC for p-Tau Ser202/Thr205 (AT8), the rabbit-specific HRP/DAB (ABC) Detection Kit (#ab64261; Abcam) was used according to the manufacturer's instructions with small adjustments. Unless otherwise indicated, all steps were performed at RT. Sections were incubated for 10 min in hydrogen peroxide blocking solution, followed by two washes in blocking buffer (1% bovine serum albumin + 1% Triton-X in PBS) for 5 min each. Then, protein block solution was applied for 10 min, and sections were washed once for 5 min in blocking buffer. Afterward, sections were incubated overnight at 4 °C with primary antibody (biotinylated AT8 [#MN1020B; Invitrogen], diluted 1:250 in blocking buffer). The next day, sections were washed four times for 5 min in blocking buffer, and the biotinylation step of the kit was left out, since the primary antibody was already biotinylated. Next, streptavidin peroxidase solution was applied for 10 min, and sections were rinsed four times in blocking buffer afterward. In the meantime, the DAB staining solution was freshly prepared by mixing 30 μl of DAB Chromogen with 1.5 ml DAB substrate. Sections were incubated with the resulting solution for 3 min under constant shaking and rinsed four times in PBS afterward. Last, sections were mounted on positively charged coverslips (#22-037-246; Superfrost Plus Microscope Slides; FisherBrand) and left at RT to dry overnight.

The next day, sections were counterstained with cresyl violet (Nissl stain). Cresyl violet was dissolved at 1 g/l in ultrapure water under stirring overnight. The next day, 2.5 ml of glacial acetic acid were added to 1 l of cresyl violet solution and stirred for 15 min at RT. The Nissl stain solution was then sterile filtered (0.22-μm filter) and heated to 50 °C. Mounted sections were stained for 10 min in prewarmed Nissl solution and then rinsed for 3 min in ultrapure water. Next, sections were destained for 10 min in 95% ethanol, followed by two 5-min incubations in 100% ethanol. In the end, sections were incubated twice for 5 min in xylene and coverslipped with CytoSeal60 (#8310-4; Thermo Fisher Scientific).

### Quantification of tau inclusions

The experimenter was blinded to mice genotype and injection paradigm during the staining and data analysis of tau inclusions and other fluorescent stains. DAB-Nissl stained sections were scanned at 20× with an Aperio Scanner (Aperio CS2; Leica Biosystems). Subsequently, sections were exported as TIFs, and all further analyses were conducted in ImageJ/Fiji (open source developed by Wayne Rasband (91)). Channels were separated into a Nissl and DAB stain image through the color deconvolution function (parameters: [r1] = 0.55554247; [g1] = 0.77908224; [b1] = 0.29052263l; [r2] = 0.2969366; [g2] = 0.5443869; [b2] = 0.78452; [r3] = 0.77666026; [g3] = 0.31092405; and [b3] = 0.54783666). Somatic inclusions in different regions were counted manually with the help of the cell counter function. Brain regions (Fig. S4) were identified based on coronal reference sections from the Allen Brain Atlas (Allen Institute for Brain Science; https://mouse.brain-map.org/static/atlas) in two standardized sections per mouse. For heat maps and somatic inclusion counts, the number of inclusions from both sections was added together when regions were present in both sections (RSA; motor cortex, primary somatosensory cortex, auditory cortex, ectorhinal and perirhinal cortex, EC, and piriform cortex). The temporal association area was included in the counts for the auditory cortex, and the amygdalar nuclei were included in the piriform cortex counts. For brain heat maps, only schematics of section 2 are shown in the main figures of the article, since most regions (except dorsal hippocampus) where somatic inclusions were scored are visible. The ipsilateral hemisphere is always displayed on the right.

To analyze the neuritic inclusion burden, regions of interests (ROIs) were drawn along anatomical regions on section's Nissl stain, applied to the thresholded DAB stain image, and the percent area within the ROI was measured. The preprogrammed “Renyi” threshold was used on all cohorts except the WT *versus* TMEM106B KO cohort, where the preprogrammed “default” threshold was used. Sections where thresholding failed were excluded from further analysis. The thresholding to measure the percent area occupied by neuritic inclusion in the hippocampus also recognized the somatic inclusions, but the area of somatic inclusions only constitutes a very small percentage of the measured total area. The number of NP tau inclusions was counted manually by searching DAB stains of APP or DKI animals for neuritic deposits surrounding circular and unstained spaces (Aβ plaques).

In addition, higher magnification images of neuritic inclusions were taken on a Zeiss AxioImager Z1 fluorescent microscope (Zeiss) with a 63× 1.4 oil objective.

### Imaging and quantification of fluorescent staining

For the *in vitro* tau seeding assay, images were taken using the automated ImageXpress Micro XLS (Molecular Devices) with a 20× air objective. Each experiment was performed in duplicate, and four images were taken per well. With ImageJ, MAP2-positive area was identified and masked over the corresponding T49 image. The percent of T49-positive area within the MAP2 mask was calculated.

Images for Aβ (D54D2) and ThioS staining were taken on a Leica DMi8 Inverted Fluorescent Microscope (Leica) with a 10× 0.25 air objective for tiled images of whole brain sections or 20× 0.4 air objective for higher magnification images of specific regions. In addition, tiled images of D54D2 and ThioS staining for hTau and DKI animals were taken on a Zeiss AxioImager Z1 fluorescent microscope (Zeiss) with a 20× 0.8 air objective. Images for GFAP and CD68 staining were taken on the same Leica microscope as mentioned previously with 20× 0.4 air objective. All image analyses were conducted using ImageJ.

For tiled images of whole brain sections stained with D54D2 antibody and ThioS, ROIs were drawn according to the ThioS image along anatomical brain regions. D54D2 and ThioS stains were thresholded separately, and the percent area occupied within each ROI was measured. For higher magnification images of D54D2 and ThioS-stained section, images were converted to 8 bit, thresholded in each channel, and the percent area was measured.

Images of cells stained for GFAP or CD68 were thresholded, followed by measuring the percent area occupied (for GFAP: objects above 15 and below 10.000 pixels were included, for CD68: objects above 5 and below 750 pixels in size were included).

### SDS-PAGE and Western blotting

All samples were heated for 5 min at 95 °C prior to loading. SDS-PAGE was performed by loading samples on 24-well 4 to 20% Criterion TGX Precast Gels (Bio-Rad) and running them in 1× Tris/glycine/SDS running buffer (#1610772; Bio-Rad) for 45 min at 180 V.

For Western blotting (WB), proteins were transferred onto nitrocellulose membranes (iBlot2 Gel Transfer Device with #IB23001, iBlot2 NC Regular Stacks; Invitrogen) and blocked for 1 h at RT with blocking buffer (#MB-070-010F, blocking buffer for fluorescent WB). Afterward, membranes were incubated overnight at 4 °C with primary antibodies (1:1000 dilution in blocking buffer, see Table 1 for antibodies). The next day, membranes were washed three times for 5 min in Tris-buffered saline + 0.1% Tween-20 detergent (TBST), followed by incubation with secondary antibodies (donkey antimouse 680 or donkey anti-rabbit 800; LI-COR Biosciences, diluted 1:10,000 in TBST) for 1 h at RT. Blots were washed again three times for 5 min in TBST and then imaged on an Odyssey Infrared Imaging System (LI-COR Biosciences). Densitometric quantification of protein bands was performed with Image Studio Lite (LI-COR Biosciences).

### Immunoprecipitation of Aβ from tau samples

Control brain and brain D tau extracts were thawed on ice, and a sample to estimate the initial tau and Aβ concentration *via* WB was taken. Pure Proteome Protein G Magnetic Beads (#LSKMAGG10; Millipore) were conjugated to either Rabbit IgG antibody (#2729; CST) or β-amyloid (D54D2) XP Rabbit mAb (#8243; CST) according to the manufacturer's instructions in PBS + 0.02% Tween-20 (PBST). For each 100 μl of tau extract, 25 μl of beads were conjugated to 1 μg of antibody. Extracts were incubated with beads overnight at 4 °C under nutation. The next day, extracts were removed from beads, transferred to a new Eppendorf tube, and a sample was taken to assess tau and Aβ concentration after clearing. Beads were washed three times in 200 μl PBS, and bound protein was eluted by incubating beads for 10 min at 95 °C with 1× Laemmli sample buffer (one-third of initial sample volume, sample E1). After this first round of immunodepletion, there was still Aβ present in the tau extracts as well as residual antibody. To further reduce the amount of Aβ in the samples, the process described previously was repeated, but with a reduced amount of antibody (one-fourth of the previous amount). After the extracts had incubated overnight with antibody-conjugated beads, the tau extracts were transferred to new Eppendorf tubes and to remove residual antibody, incubated with unconjugated Pure Proteome Protein G Magnetic Beads for 2 h at 4 °C under nutation. In the meantime, the antibody-conjugated beads were eluted once more as described previously (sample E2). After 2 h, the tau extracts were removed from the beads, and a sample was taken for analysis. The beads of the third incubation were eluted again as described previously (sample E3). The remaining tau extract was stored at 4 °C until injection into mice the next day. The decrease in monomeric and oligomeric Aβ as well as total tau was measured by densitometric analysis using ImageStudio Lite (Licor) by averaging the ratios of cleared/untreated extract band intensity per dilution.

### Statistical analysis

Figures were prepared using Adobe Illustrator CC (Adobe, Inc). All statistical analyses and graphing of data were conducted using GraphPad Prism 9 (GraphPad Software, Inc). One-way ANOVA with Tukey's multiple comparisons test, Kruskal–Wallis with Dunn's multiple comparisons test, Brown–Forsythe ANOVA test with Dunnett's multiple comparisons test or two-way ANOVA with Sidak's multiple comparisons test were performed as indicated in the legends to the figures. A *p* < 0.05 was considered statistically significant, and all values are displayed as mean ± SEM. All n values refer to individual mice unless indicated otherwise.

## Data availability

ImageJ macros and original data generated from this study are available upon request (contact corresponding author).

## Supporting information

This article contains supporting information.

## Conflict of interest

S. M. S. is an inventor on a patent application related to the use of Fyn kinase inhibitors in AD and is a cofounder and holds equity interest in Allyx Therapeutics, seeking to develop Alzheimer's therapies. The other authors declare that they have no conflicts of interest with the contents of this article.
