# Supplementary material for: Spreading of Alzheimer tau seeds is enhanced by aging and template matching with limited impact of amyloid-β
Source: J Biol Chem. 2021 Sep 2;297(4):101159. doi: 10.1016/j.jbc.2021.101159 (PMC8477193; doi:10.1016/j.jbc.2021.101159)
Supplement: Supplemental Figures S1–S4 [file mmc1.pdf]

Modulating Tau spreading in mouse models of AD

Supporting Information

Title: Spreading of Alzheimer Tau seeds is enhanced by aging and template matching with limited impact of amyloid-β

Authors: Sarah Helena Nies[1,2], Hideyuki Takahashi[1], Charlotte S. Herber[1], Anita Huttner[3], Alison Chase1, Stephen M. Strittmatter[1]

Included materials:

Supporting Figures: 4

Supporting Tables: 0

A

Internal Name	Patient Age	Post mortem Interval [h]	NIA Classification or Braak Stage	Sex
Control Brain	47	25	0	F
Brain A	87	36	A2, B3, C2	M
Brain B	87	23	A2, B3, C2	M
Brain D	64	6	VI	M

B

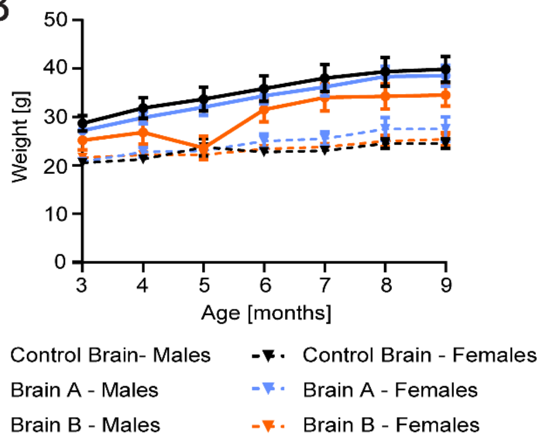

C

Cohort	Average Age at injection [months]	Injected Material	Genotype	Male (analyzed)	Female (analyzed)	Combined (analyzed)
WT vs WT cohort	3	Control	WT	6	4	10
		Brain A	WT	6	4	10
		Brain B	WT	5	5	10
		Brain D	WT	5	5	10
		Brain D conc.	WT	5	5	10
WT vs APP/PS1 cohort I	3	Control	WT	4	1	5
			APP/PS1	5 (4)	5 (0)	10 (4)
		Brain A/B	WT	5	5	10
			APP/PS1	5 (3)	5 (3)	10 (6)
WT vs TMEM KO (aged) cohort	19	Control	WT	5 (3)	5 (4)	10 (7)
			TMEM KO	3 (2)	4 (3)	7 (5)
		Brain A/B	WT	5	5 (2)	10 (7)
			TMEM KO	4 (3)	5 (4)	9 (7)
WT vs Pyk2 KO cohort	3	Control	WT	2	3	5
			Pyk2 KO	1	4	5
		Brain A/B	WT	4	2	6
			Pyk2 KO	1	4	5
WT treated with AZD	3	Brain A/B	WT - Vehicle	10	10	20
			WT - AZD	10	10	20
WT vs PGRN KO cohort	3	Control	WT	1	3	4
			PGRN KO	5	2	7
		Brain A/B	WT	3	5	8
			PGRN KO	9	3	12
WT vs APP/PS1 cohort II	3	Control	WT	3	2	5
			APP/PS1	2	2	4
		Control - D54D2	WT	3	2	5
			APP/PS1	2 (0)	2 (1)	4 (1)
		Brain D	WT	3	5	8
			APP/PS1	5 (3)	6 (5)	11 (8)
		Brain D - D54D2	WT	4	3	7
			APP/PS1	3	11 (7)	14 (10)
WT vs hTau vs hTau/NLF cohort	3	Control	WT	2	3	5
			hTau	0	4	4
			hTau/NLF	3	2	5
		Brain A/B	WT	1	3	4
			hTau	1	1	2
			hTau/NLF	3	5	8

Figure S-1: Additional information on brain tissue used for injections, animal weight and animal cohorts. A) Post-mortem information of patients whose brains were used for Tau extractions. **B)** Body weight

of Control or AD Tau extract injected animals from injection to killing. Animals were weighed once a month. **C)** Overview of injected mice cohorts. Numbers in brackets indicate the number of animals analyzed from this group.

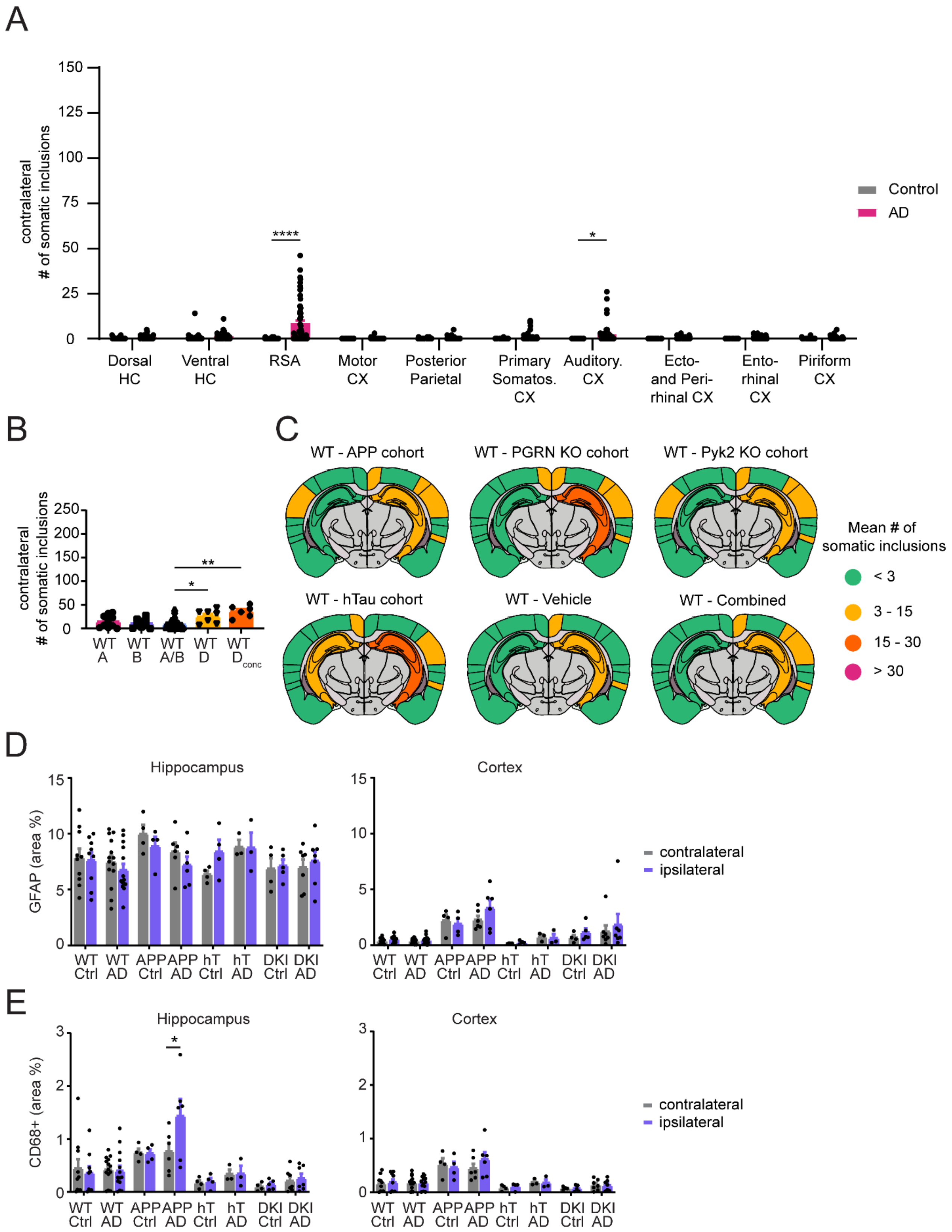

Figure S-2: Contralateral hemisphere of AD extract injected animals shows lower numbers of somatic inclusions. A) Mean number of somatic inclusions per brain region on the contralateral hemisphere in WT animals injected with Tau extracts extracted from Control or AD brains. Inclusions were counted manually in ImageJ with the Cell Counter Tool. *Statistics: Ordinary Two-way ANOVA test (Interaction: F (9, 838) = 10.01, p<0.0001; Row Factor: F (9, 838) = 10.08), p<0.0001; Column Factor: F (1, 838) = 28.13, p<0.0001) with Sidak's multiple comparisons test. N represent individual animals. N(Control)=27-28, N(AD)=60. **p<0.01, ****p<0.0001.* **B)** Mean number of somatic inclusions on the contralateral hemisphere in animals injected with Tau extracts extracted from different AD brains. *Statistics: Kruskal-Wallis test (Approximate p-value: 0.0008, Kruskal-Wallis statistic: 19.07) with Dunn's multiple comparisons. N represent individual animals. N(A)=10, N(B)=9, N(AB)=28, N(D)=7, N(D_{conc})=6. *p<0.05, **p<0.01.* **C)** Coronal section schematics of mean somatic inclusion burden in ipsi- and contralateral brain regions of WT animals injected with Brain AB Tau extract in different animal cohorts. Brain regions not analyzed are depicted in grey. Ipsilateral hemisphere is on the right. **D)** Quantification of area occupied by GFAP staining in the dentate gyrus (left) and cortex layer I-III (right) in animals injected with Control or AD Tau extracts. *Statistics: Ordinary Two-way ANOVA test (for hippocampus - Interaction: F (7, 88) = 0.6645, p=0.7014; Row Factor: F (7, 88) = 1.654, p=0.1310; Column Factor: F (1, 88) = 0.01862, p=0.8918; for cortex - Interaction: F(7, 88) = 0.5127, p=0.8228; Row Factor: F(7, 88) = 10.59, p<0.0001; Column Factor: F(1, 88) = 1.424, p=0.2360) with Tukey's multiple comparisons test to compare same hemispheres across genotypes or with Sidak's multiple comparisons test to compare ipsi- and contralateral signals of the same genotype. N represent individual animals. N(WT-C)=9-10, N(WT-AD)=14-15, N(APP-C)=4, N(APP-AD)=6-8, N(hT-C)=4, N(hT-AD)=3, N(DK-C)=4-5, N(DK-AD)=7.* **E)** Quantification of area occupied by CD68+ staining in the hippocampus (left) and cortex (right) in animals injected with Control or AD Tau extracts. *Statistics: Ordinary Two-way ANOVA test (for hippocampus - Interaction: F(7, 88) = 1.393, p=0.2185; Row Factor: F(7, 88) = 8.846, p<0.0001; Column Factor: F(1, 88) = 1.085, p=0.3005; for cortex - Interaction: F(7, 88) = 0.5283, p=0.8110; Row Factor: F(7, 88) = 13.76, p<0.0001; Column Factor: F(1, 88) = 0.2536, p=0.6158) with Sidak's multiple comparisons test to compare ipsi- and contralateral signals of the same genotype. N represent individual animals. N(WT-C)=9-10, N(WT-AD)=14-15, N(APP-C)=4, N(APP-AD)=6-8, N(hT-C)=4, N(hT-AD)=3, N(DK-C)=4-5, N(DK-AD)=7.*

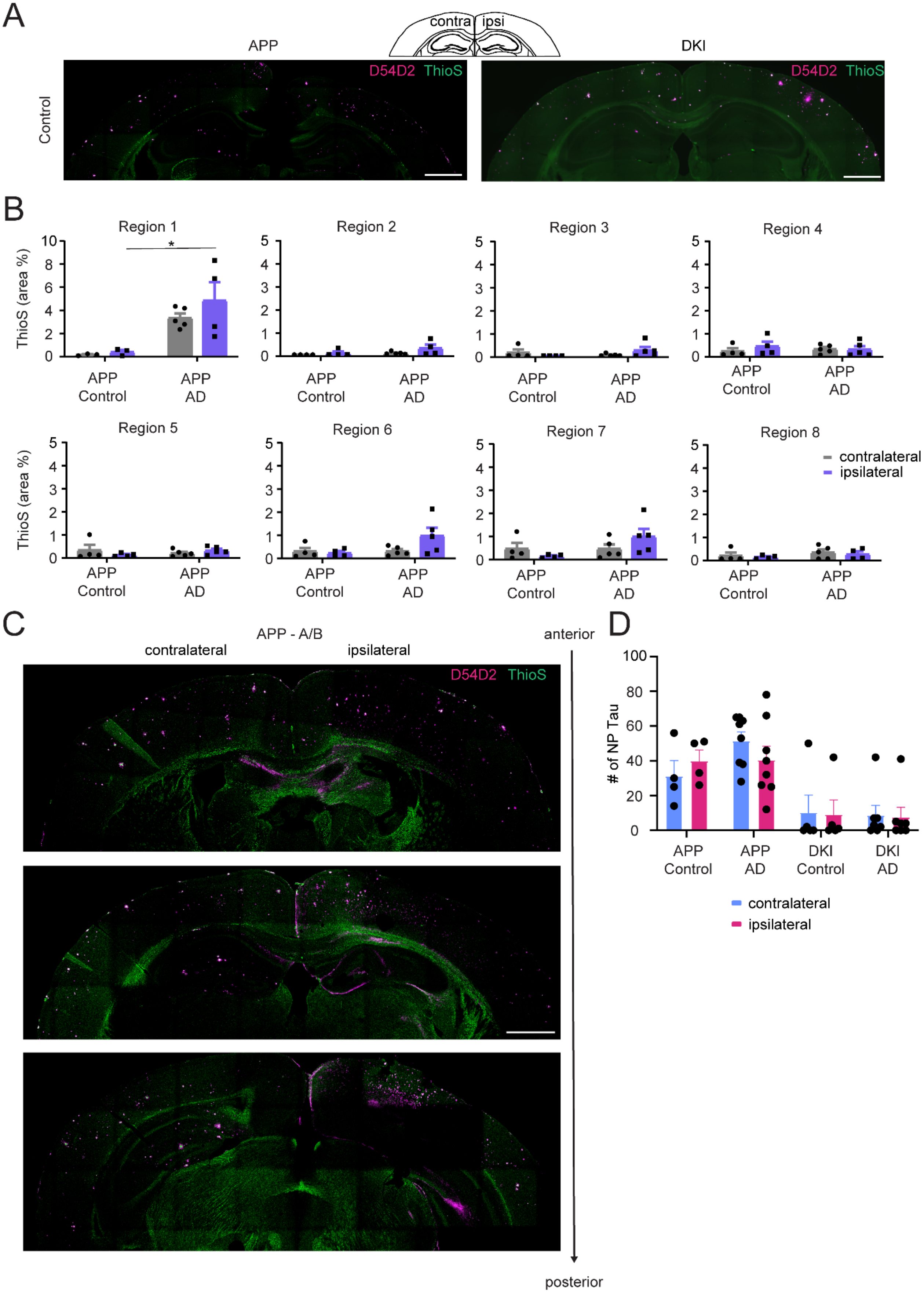

Figure S-3: Dense-core plaque Aβ does not get redistributed and ipsilateral Aβ redistributions extends anterior and posterior to the injection site. A) Representative images of immunofluorescent staining with

D54D2 (magenta) antibody for Aβ and ThioS (green) for dense-core amyloid plaques in APP and DKI mice injected with Control brain extracts. Scale bar: 1mm. **B)** Quantification of ThioS staining seen in 4D. *Statistics: Ordinary Two-way ANOVA (For Region 1 - Interaction: $F(1,11) = 0.4629$, $p=0.5103$; Row Factor: $F(1,11) = 17.11$, $p=0.0017$; Column Factor: $F(1,11) = 0.8951$, $p=0.3644$; For Region 2 - Interaction: $F(1,13) = 0.6931$, $p=0.4201$; Row Factor: $F(1,13) = 2.931$, $p=0.1106$; Column Factor: $F(1,13) = 3.046$, $p=0.1045$; Region 3 - Interaction: $F(1,14) = 2.825$, $p=0.1150$; Row Factor: $F(1,14) = 0.4270$, $p=0.5240$; Column Factor: $F(1,14) = 0.1024$, $p=0.7537$; Region 4 - Interaction: $F(1,14) = 0.6228$, $p=0.4432$; Row Factor: $F(1,14) = 0.02337$, $p=0.8807$; Column Factor: $F(1,14) = 0.5257$, $p=0.4804$; Region 5 - Interaction: $F(1,14) = 2.041$, $p=0.1750$; Row Factor: $F(1,14) = 0.07300$, $p=0.7910$; Column Factor: $F(1,14) = 0.1388$, $p=0.7151$; Region 6 - Interaction: $F(1,14) = 2.842$, $p=0.1140$; Row Factor: $F(1,14) = 2.980$, $p=0.1063$; Column Factor: $F(1,14) = 1.876$, $p=0.1924$; Region 7 - Interaction: $F(1,14) = 3.102$, $p=0.1000$; Row Factor: $F(1,14) = 3.089$, $p=0.1007$; Column Factor: $F(1,14) = 0.1771$, $p=0.6803$; Region 8 - Interaction: $F(1,13) = 0.00229$, $p=0.9625$; Row Factor: $F(1,13) = 1.370$, $p=0.2629$; Column Factor: $F(1,13) = 0.3419$, $p=0.5687$) with Sidak's multiple comparisons test comparing ipsi- and contralateral hemisphere within each injection and ipsilateral results across injections. N represents individual animals. N(APP-C)=3-4, N(APP-AD)=4-5. *$p<0.05$, **$p<0.01$.* **C)** Three coronal sections (anterior to posterior) of the same APP animal injected with AD Tau extract and stained for with D54D2 (magenta) antibody for Aβ and ThioS (green) for dense-core amyloid plaques (scale bar: 1mm). **D)** Number of neuritic plaques (NP) with AT8-positive Tau surrounding it. *Statistics: Ordinary Two-way ANOVA with Sidak's multiple comparisons test. N(APP-Ctrl)=4, N(APP-AD)=8, N(DKI-Ctrl)=5, N(DKI-AD)=7. N represents individual animals with two sections analyzed per animal.*

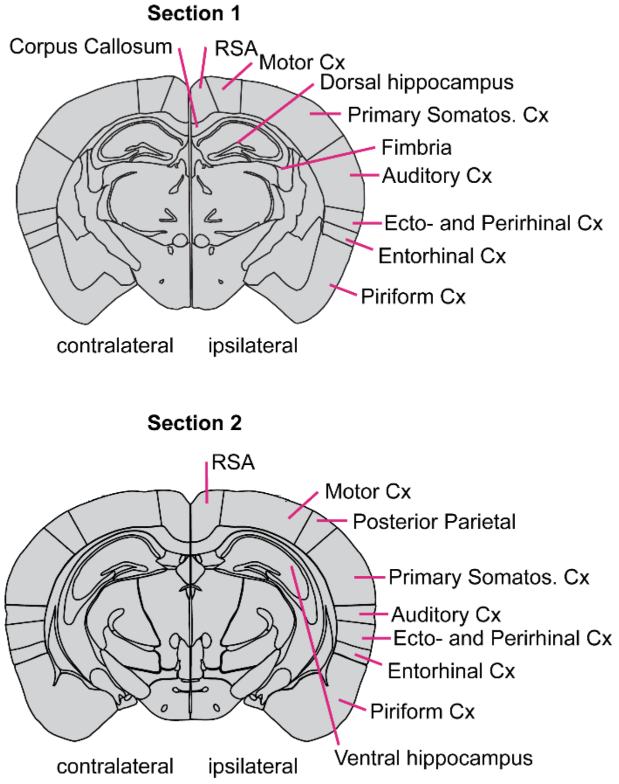

Figure S-4: Schematics of analyzed mouse brain sections. Hippocampal and cortical brain regions were identified based on coronal reference sections from the Allen Brain Atlas. Only schematics of Section 2 are shown in the main figures of the paper. Indicated regions are the ones used for counting somatic and neuritic inclusions.
